# Integrated phosphoproteomic and metabolomic profiling reveals perturbed pathways in the hippocampus of gut microbiota dysbiosis mice

**DOI:** 10.1038/s41398-020-01024-9

**Published:** 2020-10-13

**Authors:** Haiyang Wang, Lanxiang Liu, Xuechen Rao, Benhua Zeng, Ying Yu, Chanjuan Zhou, Li Zeng, Peng Zheng, Juncai Pu, Shaohua Xu, Ke Cheng, Hanping Zhang, Ping Ji, Hong Wei, Peng Xie

**Affiliations:** 1 Chongqing Key Laboratory of Oral Diseases and Biomedical Sciences, Stomatological Hospital of Chongqing Medical University, 401147 Chongqing, China; 2 College of Biomedical Engineering, Chongqing Medical University, 400016 Chongqing, China; 3 NHC Key Laboratory of Diagnosis and Treatment on Brain Functional Diseases, The First Affiliated Hospital of Chongqing Medical University, 400016 Chongqing, China; 4 Department of Neurology, Yongchuan Hospital of Chongqing Medical University, 402460 Chongqing, China; 5 Department of Laboratory Animal Science, College of Basic Medical Sciences, Third Military Medical University, 400038 Chongqing, China; 6 Department of Nephrology, The Second Affiliated Hospital of Chongqing Medical University, 400010 Chongqing, China; 7 Department of Neurology, The First Affiliated Hospital of Chongqing Medical University, 400016 Chongqing, China

**Keywords:** Depression, Molecular neuroscience

## Abstract

The dysbiosis of gut microbiota is an important environmental factor that can induce mental disorders, such as depression, through the microbiota–gut–brain axis. However, the underlying pathogenic mechanisms are complex and not completely understood. Here we utilized mass spectrometry to identify the global phosphorylation dynamics in hippocampus tissue in germ-free mice and specific pathogen-free mice (GF vs SPF), fecal microbiota transplantation (FMT) model (“depression microbiota” and the “healthy microbiota” recipient mice). As a result, 327 phosphosites of 237 proteins in GF vs SPF, and 478 phosphosites of 334 proteins in “depression microbiota” vs “healthy microbiota” recipient mice were identified as significant. These phosphorylation dysregulations were consistently associated with glutamatergic neurotransmitter system disturbances. The FMT mice exhibited disturbances in lipid metabolism and amino acid metabolism in both the periphery and brain through integrating phosphoproteomic and metabolomic analysis. Moreover, CAMKII-CREB signaling pathway, in response to these disturbances, was the primary common perturbed cellular process. In addition, we demonstrated that the spliceosome, never directly implicated in mental disorders previously, was a substantially neuronal function disrupted by gut microbiota dysbiosis, and the NCBP1 phosphorylation was identified as a novel pathogenic target. These results present a new perspective to study the pathologic mechanisms of gut microbiota dysbiosis related depression and highlight potential gut-mediated therapies for depression.

## Introduction

As the link to the second brain, the gut microbiota has an important role in regulating various pathophysiological functions in mammals. Increasing preclinical evidence indicates that the bidirectional signaling between the gut microbiome and the central nervous system, the microbiota–gut–brain axis, exerts a profound influence on brain development, function, and behavior. Gut microbiota can modulate these central processes by endocrine, immune and neural pathways within the microbiota–gut–brain axis^1,2^. Emerging research now suggests that alterations in the composition of gut microbiota (dysbiosis) are involved in psychiatric disorders such as depression, anxiety, and schizophrenia^3–6^, and neurodegenerative diseases, such as Alzheimer’s disease and Parkinson’s disease^7,8^.

Our previous studies have demonstrated that dysbiosis in gut microbiota has a causal role in the onset of major depression disorder (MDD)^6^ based on a 16S rRNA gene sequence-based approach, and there is sex-specific gut microbiota in patients with MDD^9^. We have also reported that germ-free (GF) mice, which are devoid of any bacterial contamination, showed reduced anxiety- and depression-like behaviors, along with changes in hypothalamic–pituitary–adrenal (HPA) axis^10,11^, compared with specific pathogen-free (SPF) mice. Furthermore, the colonization of GF mice with “depression microbiota” extracted from fecal samples of MDD patients resulted in increased anxiety- and depression-like behaviors as compared with mice colonized with “healthy microbiota” from healthy controls^6,12^. Through fecal microbiota transplantation (FMT), gut microbiome remodeling mice can be used as an animal model to explore the pathogenic mechanisms of disorders, such as depression^13,14^.

To provide insight into the mechanisms of host–microbiota interactions, omics technologies have been widely used to detect molecular alterations in peripheral and central samples of rodents^15–17^. MicroRNA and messenger RNAs expression disturbances in the hippocampus of GF mice, detected by microarray and real-time polymerase chain reaction (PCR) analysis, demonstrated that the axon guidance, glucocorticoid receptor pathway, and PKC–CREB signaling pathways were significantly perturbed^10,11,18,19^. In addition, Metabolic changes in the liver, cecum, serum, and hippocampus of FMT mice, measured by a metabolomics approach, suggested that dysbiosis of the gut microbiome may have a causal role in the development of depression via modulating host metabolism (e.g., lipid and energy metabolism, amino acid metabolism)^6,12^. However, the underlying mechanisms mediating gut microbiota effects on brain functions are complex and still remain obscure.

Post-translational modifications (PTMs), such as phosphorylation, are crucial regulators of protein functions and signaling and can be used as important instruments to understand the molecular pathways and signaling networks in pathological processes. In order to obtain a more comprehensive picture of the microbiota–brain axis, we used global phosphoproteomic analysis of hippocampus tissue by the tandem mass-tag (TMT) labeling combined with liquid chromatography–tandem mass spectrometry (LC–MS/MS) to identify dysregulated protein phosphorylation-dependent signaling and other biological processes disrupted in GF and FMT models.

## Materials and methods

### GF mice

Eight-week-old male GF (*n* = 8, 30–40 g) and SPF (*n* = 8, 30–40 g) Kunming mice (research resource identifier (RRID): MGI: 5651867) were provided by the Experimental Animal Research Center at Third Military Medical University (Chongqing, China). GF mice were housed in flexible film gnotobiotic isolators, while the SPF mice were housed in the standard animal facility. All experimental mice were kept under the same standard environmental conditions less than 60 dB (12-h light-dark cycle with lights on at 08:00–20:00; constant temperature, 23 ± 1°C; relative humidity, 50 ± 5%). The care and use of animals for experimental procedures were in accordance with the guidelines laid down by the NIH and approved by the Ethics Committee of Chongqing Medical University (Chongqing, China; 2017013). This study was not pre-registered and no sample calculation was performed.

### FMT mice

The FMT model was established as described in the previous studies^6,20^. Briefly, adult male GF mice (8 weeks) were colonized with microbiota from fecal samples of five MDD patients and five healthy controls, respectively. A total of 0.5 g fecal samples (0.1 g from each individual), obtained from MDD patients or healthy controls, were mixed in 7.5 ml of 0.9% sterile saline to obtain suspensions. Then, the GF mice were randomly colonized with pooled suspensions derived from either MDD patients or healthy controls. To prevent the normalization of gut microbiota, the “depression microbiota” (*n* = 6) and the “healthy microbiota” recipient mice (*n* = 6) were separately housed in different gnotobiotic isolators under the same standard environmental conditions.

### Sample collection and preparation

After 2 weeks, the GF, SPF, and FMT mice were sacrificed with minimal pain after anesthesia with chloral hydrate (200 mg/kg) and hippocampus samples were collected rapidly, snap-frozen in liquid nitrogen, and stored at −80 °C until further use. Samples were grinded 30 min by liquid nitrogen, then the powders were transferred to 5 mL centrifuge tubes and sonicated three times on ice using a high-intensity ultrasonic processor (Scientz) in lysis buffer (8 M urea, 2 mM EDTA, 10 mM DTT, 2% phosphatase inhibitor cocktail V and 0.1% protease inhibitor cocktail). Total protein concentration was determined by the 2-D Quant kit according to the manufacturer’s instructions. For trypsin digestion, the protein samples were diluted with 100 mM TEAB and further digested with trypsin (1:50 w/w) for the first digestion overnight and (1:100 w/w) for the second 4 h-digestion. The reaction was quenched with 1% formic acid and followed by peptide desalting with Strata X C18 SPE column (Phenomenex) and vacuum-dried. Peptides were reconstituted and processed with 6-plex TMT kit.

### HPLC fractionation and phosphopeptide enrichment

The sample was then fractionated into fractions by high pH reverse-phase HPLC using Agilent 300Extend C18 column (5 μm particles, 4.6 mm ID, 250 mm length). Phosphopeptides were further enriched by IMAC microspheres. To elute the enriched phosphopeptides from the IMAC microspheres, an elution buffer containing 10% NH_4_OH was added and the enriched phosphopeptides were eluted with vibration. The supernatant containing phosphopeptides was collected and lyophilized for LC–MS/MS analysis.

### Liquid chromatography–mass spectrometry identification

Enriched phosphopeptide mixtures were dissolved in solvent A (0.1% FA in 2% ACN), directly loaded onto a reversed-phase pre-column (Acclaim PepMap 100, ThermoScientific) and separated on a reversed-phase analytical column (Acclaim PepMap RSLC, ThermoScientific) with a linear gradient of 4–22% solvent B (0.1% FA in 98% ACN) for 50 min, 22–35% solvent B for 12 min, 35–85% solvent B for 4 min and holding at 85% for the last 4 min at a constant flow rate of 300 nL/min on an EASY-nLC 1000 UPLC system. The resulting peptides were analyzed by Q Exactive^TM^ Plus hybrid quadrupole-Orbitrap mass spectrometer (ThermoFisher Scientific). The pooled protein samples were detected with three technical replicates.

The resulting MS/MS data were processed using MaxQuant with an integrated Andromeda search engine (v.1.4.1.2). Carbamidomethylation on Cys was specified as fixed modification and oxidation on methionine, phosphorylation on serine (Ser), threonine (Thr), tyrosine (Tyr), and acetylation on protein N-terminal were specified as variable modifications. False discovery rate (FDR) thresholds for candidate protein, peptide, and phosphorylation site were specified at 1%.

### Bioinformatics analysis

The fold-change ratio and *p*-value were calculated for each phosphorylation site. Cutoff values of fold-change above 1.5 and below 0.67 with *p*-value < 0.05 were considered to be upregulated and downregulated, respectively. Significantly dysregulated phosphopeptides between the two experimental conditions were investigated for consensus sequences with the MoMo software tool using Motif-X algorithm^21^. Ser or Thr was set as central residue with a window width of 13, the minimal occurrence was set to 20, and significant was 0.000001. Identified motifs were then investigated for predicted upstream kinases using PhosphoMotif Finder^22^. To further understand the function and feature, gene ontology (GO)^23^ annotations were performed on the significant phosphoproteins. Then, these phosphoproteins were mapped onto the KOBAS web server^24^ and Ingenuity Pathway Analysis software (IPA) to identify functional pathway and network perturbances. Moreover, the STRING database^25^ was used to analyze the protein–protein interactions (PPI) of common phosphoproteins to explore novel pathogenic targets. High-quality interactions were extracted and displayed in Cytoscape^26^. In addition, an integrated analysis of the present phosphoproteomic results and our previous metabolomic data in FMT mice^6^ was performed to further identify and characterize the metabolic mechanisms of gut microbial dysbiosis caused by depression. In order to identify robust brain functional alterations in depression, we compared the phosphoproteomic profiling of the FMT mice to that of stress-induced depression rats^27^ and MDD postmortem brains^28^.

## Results

### Behavioral phenotypes

FMT experiments have been successfully used to explore the causative role of gut microbiota in intestinal inflammatory diseases^29^, diabetes^30^, and obesity^20,31^. In the present study, this approach was used to investigate the effects of gut microbiome alterations in the pathogenesis of depression. As previously described^6^, GF mice exhibited significantly decreased immobility time in the forced swimming test (FST) and increased center distance proportion in the open field test (OFT) relative to SPF mice, indicating reduced depression-like and anxiety-like behaviors, respectively. In contrast, as compared to the FMT-HC mice, the FMT-MDD mice displayed significant depression-like and anxiety-like behaviors as evidenced by an increased immobility time in the FST and a decreased center distance proportion in the OFT, respectively. The experimenters were unaware of the animal’s group during behavior tests.

### Comparative phosphoproteome analysis

**Fig. 1:**
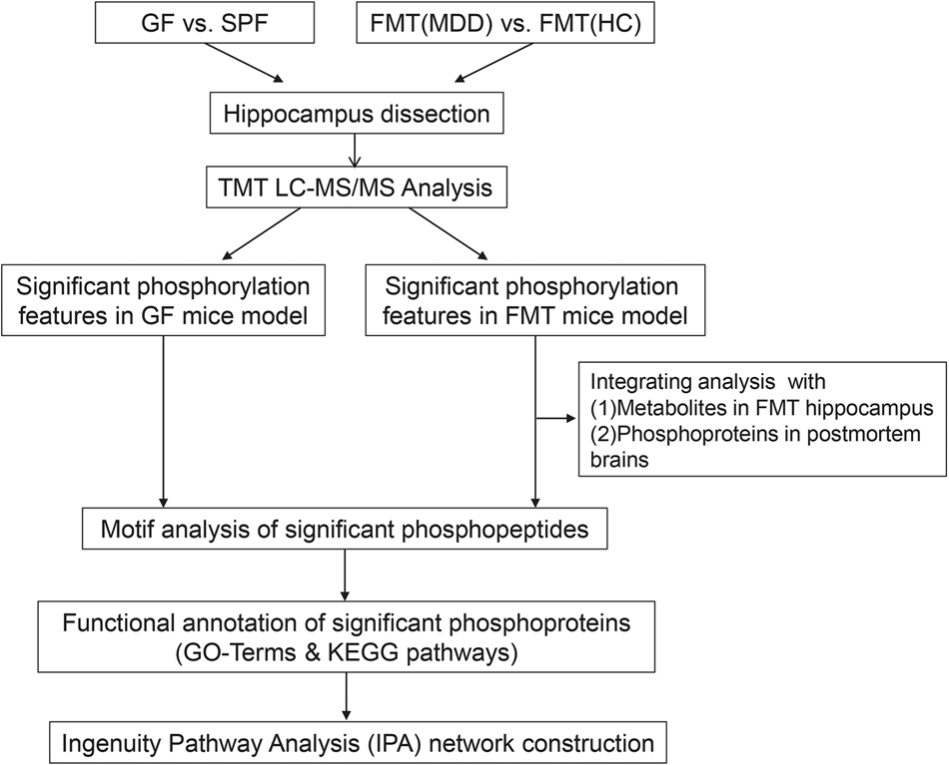
Stepwise experimental flowchart depicting the analysis of this study. GF germ-free, SPF specific pathogen-free, FMT-MDD “depression microbiota” recipient mice, FMT-HC “healthy microbiota” recipient mice.

Both the deficiency and alterations in the composition of commensal microbiota are types of dysbiosis. Thus, we performed global quantitative phosphoproteomic profiling on three replicates of hippocampus tissues from GF and FMT models to obtain a comprehensive understanding of hippocampal signaling cascades impacted by gut microbial dysbiosis. A stepwise experimental flowchart depicting the analysis of this study was shown in Fig. 1. Initial analysis between GF and SPF mice revealed a large number of proteins appeared phosphorylation dysregulation. In total, 6945 phosphorylation sites in 2370 proteins were identified. We observed mostly Ser phosphorylation (86.6%), followed by Thr (12.4%) and Tyr (1.0%) (Supplementary Fig. S1A), indicating a preference for Ser phosphorylation. In the FMT model, a total of 6787 phosphorylation sites in 2580 proteins were identified. The percentages of phosphorylation sites mapped to Ser, Thr, and Tyr residues were similar to that of GF vs SPF comparison (Supplementary Fig. S1B). Analysis of the number of modified sites within each phosphoprotein revealed that more than half of the phosphoproteins contained multiple sites (Supplementary Fig. S1C, D). Based on the fold-change cutoff (above 1.5 or below 0.67) and *p*-value < 0.05, 28 phosphosites in 25 proteins are upregulated and 299 phosphosites in 216 proteins are downregulated in GF mice compared to SPF mice (Supplementary Table S1). Moreover, upregulated and downregulated phosphosites were concurrently detected in 4 phosphoproteins. Between FMT-MDD and FMT-HC mice, 103 phosphosites in 81 proteins are upregulated and 375 phosphosites in 259 proteins are downregulated (Supplementary Table S2), and 6 phosphoproteins were characterized as upregulated and downregulated in different phosphosites.

### Phosphorylation motif analysis

To further investigate the essential nature of the phosphorylation sites and identify the potential consensus motifs, the occupancy frequency of the amino acid residues in the positions surrounding the identified Ser and Thr sites were examined based on the significant phosphopeptides. In the GF model, four Ser phosphorylated consensus motifs along with kinases predicted to target these sequences were detected (Supplementary Table S3). The consensus sequence Rxxs targeted by CaMKII, PKA, and PKC were identified on 105 significant phosphopeptides. In addition, three Ser and one Thr phosphorylated consensus sequences (i.e., sP, Rxxs, sxxE, and tP) were detected in the FMT model (Supplementary Table S3). Proline located in the +1 position of Ser sites was the most conserved residues, and this consensus sequences targeted by GSK-3, ERK1/2, and CDK5 were identified on 187 significant phosphopeptides. These results suggested that the gut microbiota dysbiosis may influence brain function through phosphorylation in these conserved residues by these kinases.

### Phosphoprotein GO annotation and KEGG pathway analysis

**Fig. 2:**
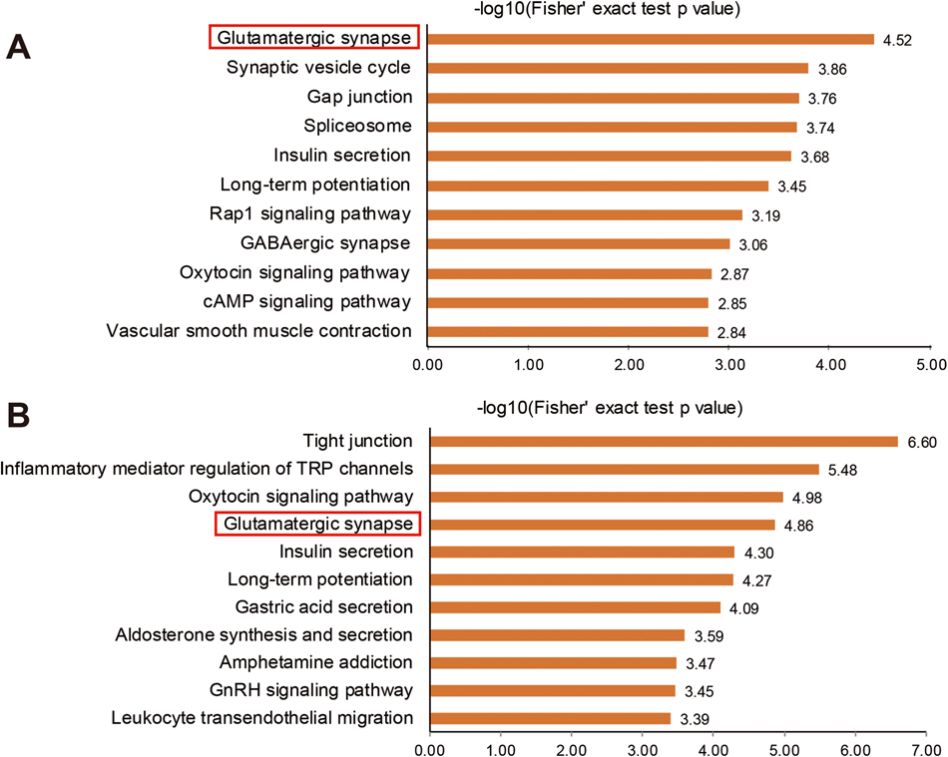
Functional pathways analysis for the significant phosphoproteins using the KOBAS web server based on the KEGG database. **a** Functional pathways perturbed in GF vs SPF. **b** Functional pathways perturbed in FMT-MDD vs FMT-HC.

To functionally categorize these significant phosphoproteins, we used GO analysis for annotation and visualization. The results of the GF model showed that the most significant GO terms were binding (Supplementary Fig. S2A), cellular process (Supplementary Fig. S2B), and cell (Supplementary Fig. S2C) for molecular function, biological process, and cellular component, respectively. These results were similar to those of the FMT model (Supplementary Fig. S2D–F). Subcellular location annotation of these phosphoproteins was also analyzed for both GF and FMT models (Supplementary Fig. S3A, B). Over half of the phosphoproteins were located in the nucleus, indicating dysregulation in the phosphorylation of nuclear signaling cascades impacted by gut microbial dysbiosis. Systematic pathway analysis for the significant phosphoproteins in the GF model was illustrated in Fig. 2a. Protein phosphorylation regulates a wide range of cellular functions. Glutamatergic synapses were the most significant prominently perturbed functional element, followed by a gap junction. Interestingly, spliceosome, never directly implicated in gut microbial dysbiosis before, was identified as a significant perturbed function in this result (Supplementary Fig. S4). Moreover, the significant phosphoproteins in the FMT model were mainly associated with the dysfunction of cell tight junctions, as well as the disturbances in the inflammatory response and glutamatergic synapses (Fig. 2b). However, microbial colonization of GF mice is always associated with a flare of transient inflammation locally and systemically. These results suggested that the gut microbiota dysbiosis per se may influence brain function through neurotransmitter disturbances.

### IPA pathway and network construction

**Fig. 3:**
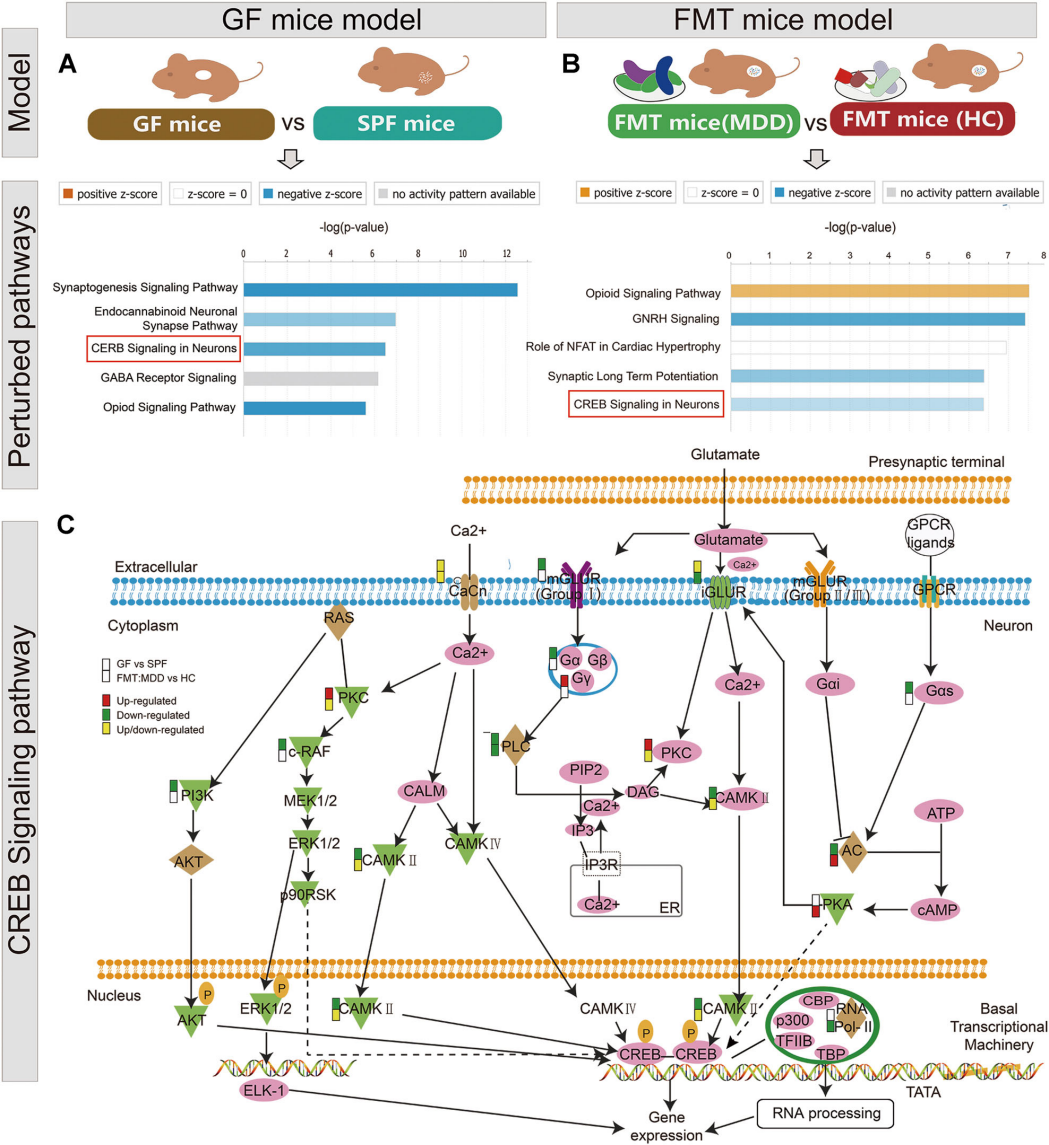
Functional network construction for the significant phosphoproteins using Ingenuity Pathway Analysis (IPA) software. **a** Perturbed pathways in germ-free (GF) mice model. **b** Perturbed pathways in fecal microbiota transplantation (FMT) mice model. **c** The visualization of CREB signaling pathway alterd significantly in GF and FMT mice models.

To enhance the reliability of the enrichment results, functional pathway analysis was performed using IPA software (Fig. 3). Synaptogenesis signaling pathway was the top perturbed canonical pathway in GF mice. And, gene expression, protein synthesis, cancer were the most significantly disturbed functions (Supplementary Fig. S5). Consistently, the CREB signaling cascade in neurons was identified as the top overlapping perturbed canonical pathway between GF and FMT models. Phosphorylation of CREB can be caused by CAMKII in response to glutamate and activated CREB results in the recruitment of transcriptional coactivators, and subsequently leads to gene expression. Moreover, these differential phosphoproteins in FMT mice were mainly related to cancer, organismal injury, and abnormalities, reproductive system disease (Supplementary Fig. S6). Interestingly, the factors including HNRNPA0, HNRNPUL2, GEMIN5, NCBP1, and NCBP3 are the building blocks of the spliceosome, which has an important role in RNA processing. Taken together, these results indicate the disturbance of the CREB signaling pathway and RNA processing function with gut microbial dysbiosis.

### Common phosphoproteins analysis

**Fig. 4:**
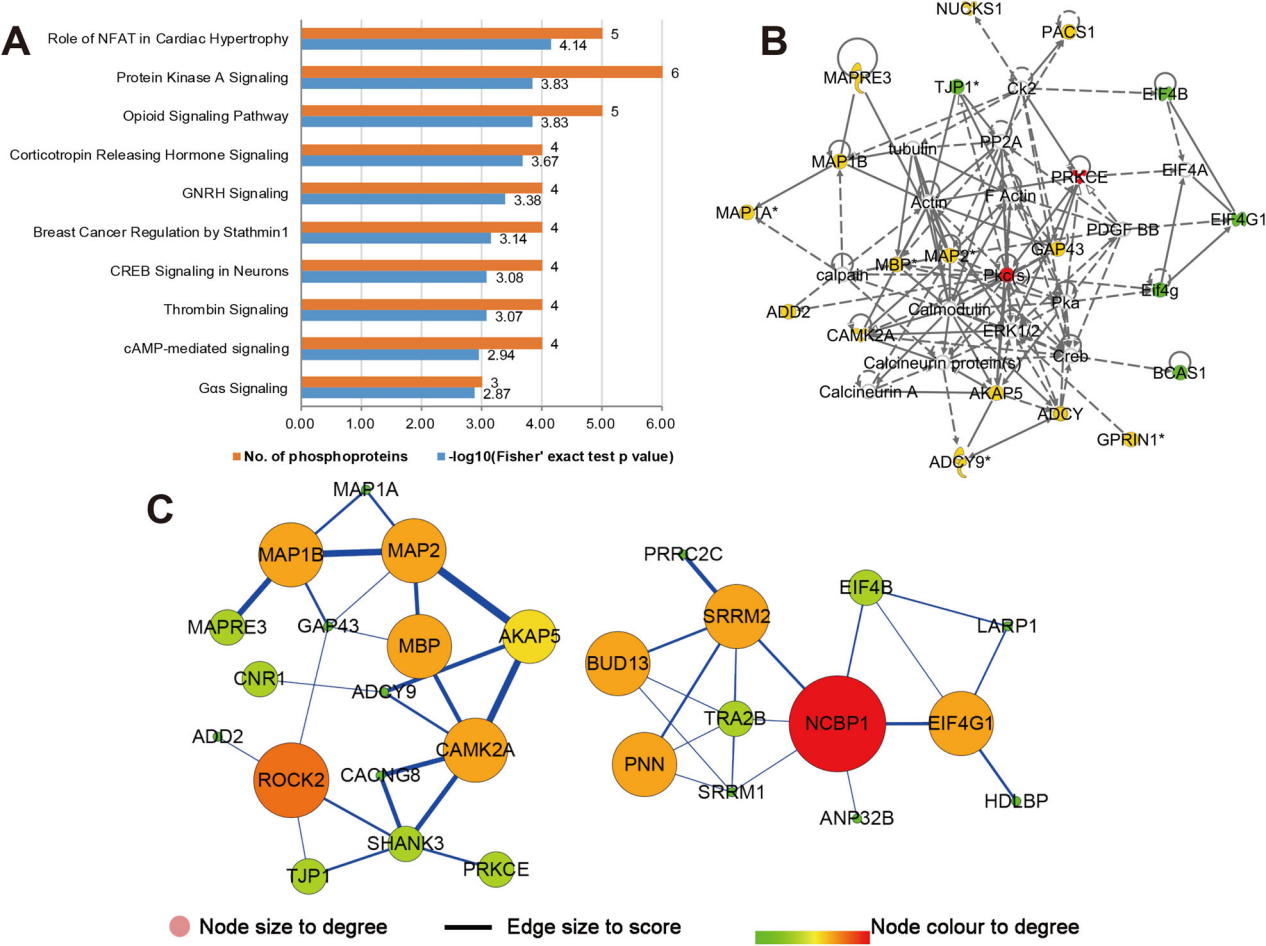
Bioinformatics analysis for the microbial-specific phosphoproteins. **a** Canonical pathway analysis for the common phosphoproteins between GF vs SPF and FMT-MDD vs FMT-HC comparisons using Ingenuity Pathway Analysis (IPA) software. **b** The most significantly disturbed functional network resulting from IPA. **c** Protein–protein interaction network analysis for these common phosphoproteins.

The absence of gut microbiota and the changes of microbial consortia structure all belong to one of the subtypes of gut microbiota dysbiosis. To determine the common perturbed functions involved in different subtypes of gut microbiota dysbiosis. We found 46 common phosphoproteins when overlapping their phosphorylation levels with GF vs SPF and FMT-MDD vs FMT-HC comparison. (Supplementary Table S4). Fourteen phosphoproteins (in bold) have the same phosphorylation sites between GF and FMT models. Common phosphoproteins analysis indicates that phosphorylation of these proteins is an important way for gut microbiota to regulate brain function and behavior. Significantly, PKA signal transduction, the upstream activator of CREB, was the second-ranking disturbed canonical pathway with the most phosphoproteins (i.e., ADCY9, ADD2, AKAP5, CAMK2A, PRKCE, and ROCK2) mapped to it (Fig. 4a). And these common phosphoproteins were mainly involved in dysfunctions of cellular assembly and organization, cellular function and maintenance, cellular development (Fig. 4b). Moreover, a total of 28 proteins and 43 direct interaction edges were included in the PPI network (Fig. 4c). Functions of the proteins in the left panel are mainly associated with inflammatory mediator regulation of TRP channels, and these in the right panel are RNA processing. Interestingly, NCBP1, with the most significant interaction degree, was recognized as the hub protein and may serve as a novel-specific pathogenic target by which gut microbiota dysbiosis affects brain function and behavior.

### Integrated phosphoproteomic and metabolomic analysis in FMT mice

Unbiased metabolomic results of serum and hippocampus were re-analyzed to identify the specific peripheral and central responses associated with the pathophysiology of gut microbial dysbiosis. The differential metabolites are listed in Table 1 and details in the previous reports^6^. These significant peripheral and central disturbances indicate that gut microbial dysbiosis may induce disorders through pathways mediated by the host’s lipid and amino acid metabolism. Moreover, lipid compositional changes can affect membrane function and subsequently affect various cellular signaling processes through the membrane—G protein and/or ion channels—cytoskeleton signal transmission pathways^32,33^. Interestingly, various subtypes of voltage-dependent Ca^2+^ channels (e.g., CACNB1, CACNG4, and CACNG8) and glutamate ionotropic receptors (e.g., GRIN2B), as the upstream molecules of CAMKII-CREB signaling pathway (Fig. 4a), exhibited dysregulated phosphorylation. In addition, glutamatergic neurotransmitter disturbance was the important alterations in FMT mice.

**Table 1:** Differential serum and hippocampal metabolites between FMT-MDD and FMT-HC mice.

Metabolites	Fold change (con/dep mice)	Changes (dep/con mice)	*p*-value	Metabolic pathways
*Serum*				
Glutathione	0.63	Down	2.46E−02	Amino acid metabolism
Pyroglutamic acid	0.48	Down	7.27E−03	Amino acid metabolism
l-Threonine	0.56	Down	5.55E−03	Amino acid metabolism
d-Fructose	−0.58	Up	4.84E−03	Carbohydrate metabolism
Palmitic acid	−0.49	Up	7.22E−03	Lipid metabolism
l-Carnitine	−1.11	Up	4.14E−06	Lipid metabolism
Linoleic acid	−0.7	Up	2.17E−02	Lipid metabolism
Oleic acid	−0.77	Up	2.19E−02	Lipid metabolism
Arachidonic acid (peroxide free)	0.47	Down	1.88E−02	Lipid metabolism
Cholesterol	1.3	Down	2.15E−02	Lipid metabolism
Stearic acid	0.28	Down	1.49E−02	Lipid metabolism
2-Hydroxyhexadecanoic acid	0.58	Down	3.96E−03	Lipid metabolism
*cis*-7-Hexadecenoic acid methyl ester	0.87	Down	1.82E−02	Lipid metabolism
Glycerol-3-phosphoric acid	−1.25	Up	9.47E−04	Not available
PC(13:0)	−0.36	Up	1.79E−02	Not available
Glycerol-2-phosphoric acid	−0.5	Up	1.12E−02	Not available
Indolelactic acid	0.52	Down	3.20E−02	Not available
PGA2 methyl ester	0.4	Down	9.58E−03	Not available
Hydroxyprogesterone acetate	0.21	Down	3.78E−02	Not available
Veratric acid	0.66	Down	5.41E−03	Not available
Sebacic acid	0.46	Down	2.75E−02	Not available
l-Threonic acid	0.53	Down	3.74E−02	Not available
*Hippocampal*				
l-Asparagine	−1.72	Up	7.73E−04	Amino acid metabolism
*N*-acetyl-l-aspartic acid	0.41	Down	4.99E−03	Amino acid metabolism
Glycine	0.17	Down	2.24E−02	Amino acid metabolism
Phenylalanine	0.41	Down	5.88E−03	Amino acid metabolism
Leucine	0.52	Down	8.03E−03	Amino acid metabolism
α-d-Glucose	−0.75	Up	5.98E−04	Carbohydrate metabolism
d-Lactose	−0.89	Up	4.99E−04	Carbohydrate metabolism
Malic acid	−0.71	Up	1.61E−03	Carbohydrate metabolism
Linolenic acid ethyl ester	−0.13	Up	2.49E−02	Lipid metabolism
Myristic acid	−0.26	Up	2.01E−02	Lipid metabolism
Palmitic amide	−0.17	Up	6.82E−03	Lipid metabolism
Ricinoleic acid methyl ester	−0.26	Up	5.39E−03	Lipid metabolism
1-Monopalmitin	−0.24	Up	2.64E−03	Lipid metabolism
*N*-palmitoylsphingosine	0.18	Down	4.43E−01	Lipid metabolism
Phytosphingosine	−0.13	Up	2.42E−02	Lipid metabolism
PGF2α dimethyl amide	−0.5	Up	2.61E−04	Not available
dihydrotachysterol	−0.13	Up	4.02E−02	Not available
Glucoheptonic acid	−0.79	Up	1.31E−04	Not available
β-Hydroxy-β-methylglutaric acid	0.28	Down	2.86E−02	Not available

### Integrated phosphoproteomic analysis of depressive rodent and human brains

**Fig. 5:**
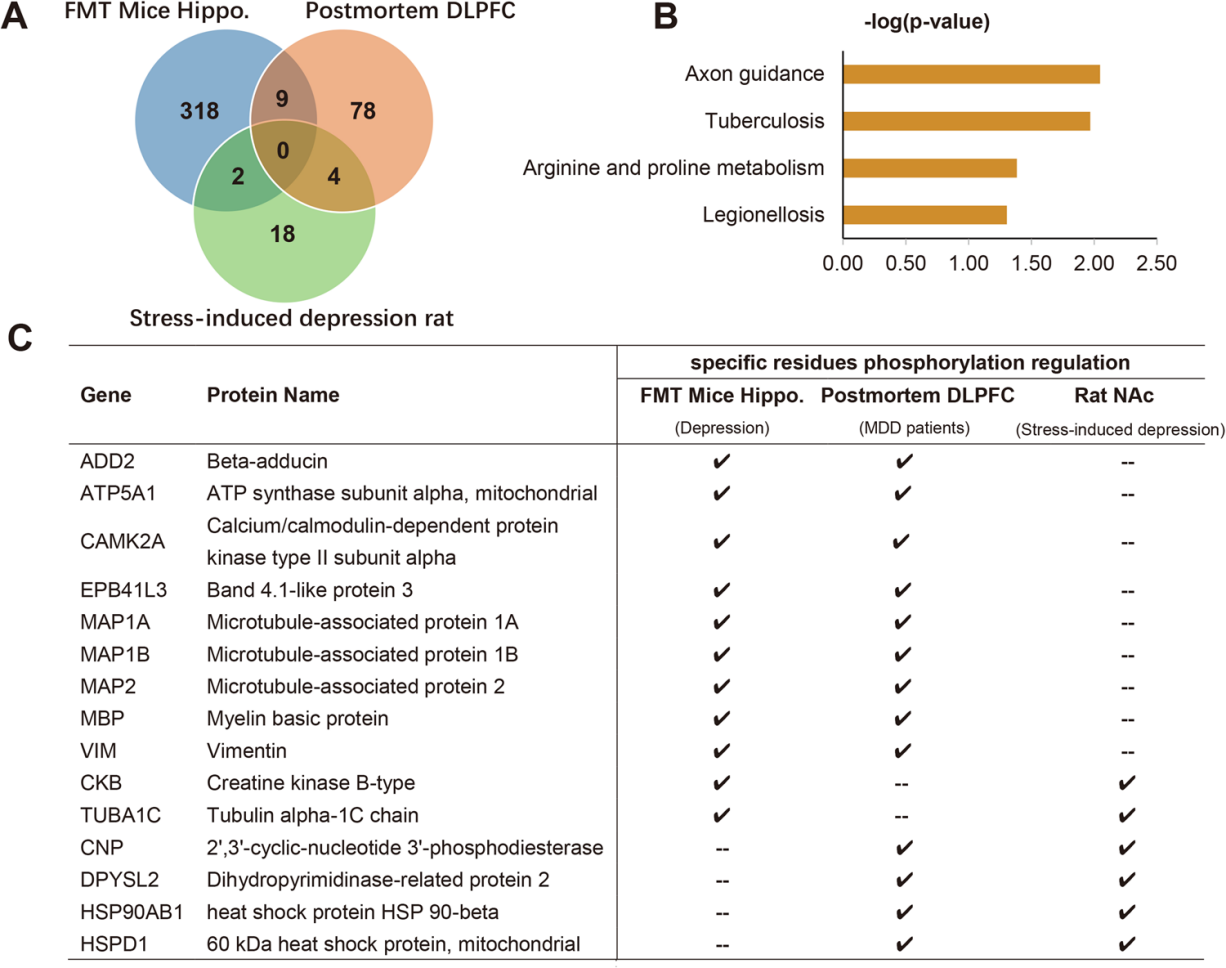
Integrated phosphoproteomic analysis of depressive rodents and patients with major depression disorders. **a** Venn diagram showing the number of overlapped and specific phosphoproteins across depressive mice, rats, and patients with major depression disorder. **b** Detailed information of the overlapped phosphoproteins identified as significantly changing across all phosphoproteomic screens. **c** Functional enrichment analysis results for all these overlapped phosphoproteins using KOBAS web server.

Increasing evidence shows that stress precipitate depressive reactions and contributes to the pathogenesis of major depression^34^. Interestingly, Gut microbes changed significantly after stress^35^. In order to identify robust brain functional alterations in depression, we compared the phosphoproteomic profiling of the FMT mice to that of stress-induced depression rats^27^and MDD postmortem brains^28^ (Fig. 5a). Finally, we found a total of 15 phosphoproteins were common between the hippocampus from FMT depressive mice, postmortem dorsolateral prefrontal cortex (DLPFC) tissue from MDD patients and the nucleus accumbens (NAc) from stress-induced depression rats (Fig. 5b). Functional enrichment analysis for all these common phosphoproteins revealed that axon guidance was the primary altered functional pathway (Fig. 5c). Previous study has documented that the diversity in axon guidance receptors for neuronal development can be generated by alternative pre-mRNA splicing with a spliceosome complex^36^. And the presence and functionality of several spliceosome-related components in neuronal dendrites suggested that the dendritic splicing may be related to synapse function^37^. Thus, we concluded that gut microbiota dysbiosis may influence synaptic function through pre-mRNA processing, eventually leading to changes in brain function and behavior.

## Discussion

The peripheral system could exert remote control over brain health and disease through the periphery–brain communication, mediated by neural, endocrinal, metabolic, and immune pathways^38^. Increasing evidence supports the potential role of microbiota–gut–brain axis in mental disorders (e.g., depression). Thus, the present key task was to gain insight into the biological basis of host–microbial interactions. In this study, we used two weeks post-FMT as the benchmark time point for assessing disease-related phenotypes according to previous studies^39,40^. As result, we demonstrated that the GF and FMT models exhibited global dysregulation in protein phosphorylation. These phosphorylation dysregulations were consistently associated with glutamatergic neurotransmitter system disturbances. The FMT-MDD mice, which can serve as an animal model of depression, exhibit disturbances in lipid metabolism and amino acid metabolism in both the periphery and brain. Furthermore, the CAMKII-CREB signaling pathway, in response to these disturbances, was the primary common cellular process identified in the GF and FMT models. In addition, the spliceosome, never directly implicated in mental disorders before, was a significantly perturbed neuronal functional element affected by gut microbial dysbiosis, and the NCBP1 phosphorylation was inferred as a novel-specific pathogenic target. These results highlight the pathologic mechanisms underlying gut microbiota dysbiosis related depression.

The relationship between lipid metabolism and gut microbiota is important for host physiology and disorders. We observed that the gut microbiota dysbiosis disturbed the levels of fatty acids and their derivatives. Interestingly, the ester of l-carnitine, acetyl-l-carnitine, is critical for hippocampal function^41,42^. Omega-6 polyunsaturated fatty acids, which act as the precursors for both pro- and anti-inflammatory mediators^43^ and have a central role in the function of the brain, were altered. Similar alterations were identified in our previous study^44^. Consistently, previous findings have indicated that sphingomyelin metabolism perturbances could impact the excitability of hippocampal neurons^45^. Changes in the composition of lipids can affect membrane stabilization, which could influence connections between the membrane and the cytoskeleton through interactions with the G protein and ion channels, subsequently altering various other cellular signaling processes^32,33^. In the present results, the phosphorylation of various subtypes of voltage-dependent Ca^2+^ channels, glutamate ionotropic receptors, K+ channels, Na+ channel, and K+/Na+ channel was dysregulated. Several prior studies have implicated the dysfunction of these channels in the pathophysiology of depression^46–48^ and other psychiatric disorders^49,50^. Moreover, PTMs help to regulate function and stability of cytoskeletons, abnormal phosphorylation of numerous cytoskeletal proteins were observed in the present study, and these abnormalities may induce changes in dendritic morphology in depression^51^. Taken together, these results indicate that gut microbial dysbiosis may induce depression by affecting host lipid metabolism and cellular signaling processes.

It has recently been reported that gut microbiota affects host brain functions by disrupting amino acid metabolism^52^. Several amino acids were altered under gut microbiota dysbiosis states, of which, glutathione, pyroglutamic acid, l-asparagine, *N*-acetyl-l-aspartic acid, and glycine are associated with glutamate metabolism—a recognized hypothesis of depression^53^. Furthermore, gut microbiota could influence the production and absorption of neurotransmitters (e.g., glutamate and GABA), increasing their bioavailability to the central nervous system^54^. Interestingly, glutamatergic and GABAergic signaling pathways were identified as significantly altered functions in the present phosphoproteins enrichment analysis. GRIN1 and GRIN2A, the subunits of NMDA receptors, exhibited dysregulated phosphorylation. Metabotropic glutamate receptor (GRM5) was downregulated at Ser851 residue phosphorylation. Phosphorylation at Thr2 and Ser4 in SLC1A3, a subunit of glutamate transporters, were downregulated in GF mice. These changes are responsible for dysfunction in neuronal excitability and synaptic transmission^55^. Moreover, phosphorylation of glutamate decarboxylase (GAD) at Ser55 was downregulated in GF mice, and the PTMs of GAD affect its activity and are responsible for synthesis turbulence of GABA for neurotransmitter functions^56^. Disturbance in the GABAB receptor subunit 1 (GABBR1) phosphorylation could affect GABA neurotransmitter function. In addition, G protein gamma subunits and adenylyl cyclase, which contribute to cellular signaling cascades, exhibited dysregulated phosphorylation in the present result, and these changes can influence the release of glutamate and GABA as neurotransmitters^57,58^. Thus, we hypothesize that gut microbiota dysbiosis may affect brain functions through the release and absorption of glutamate and GABA neurotransmitters, characterized by dysregulated phosphorylation in associated proteins.

The dysregulation of phosphorylation in postsynaptic glutamate receptors, as well as in voltage-dependent Ca^2+^ channels, could change the intracellular Ca^2+^ and disrupt the activities of CAMKII-CREB signaling pathway^59^. CREB is a transcription factor, and its interaction with BDNF has a critical role in the altered neuroplasticity observed in major depression. Consistently, our previous iTRAQ-based proteomics analysis reported a disruption of the CAMKII-CREB-BDNF signaling pathway in gut microbiome remodeling mice^60^, and p-CREB was decreased in the hippocampus of chronic unpredictable mild stress exposed mice^61^. In addition, increased p-CREB is related to treatment response in MDD patients^62^. These findings suggest that the CAMKII-CREB signaling pathway is involved in the pathogenesis of gut microbiota dysbiosis associated disorders.

Pre-mRNA splicing is catalyzed by complex cellular machineries, spliceosomes, which are composed of five uridine-rich small nuclear RNAs (the U1, U2, U4, U5, and U6 snRNPs) and numerous proteins^63^. The spliceosome has been recognized as a target of novel antitumour drugs through altering gene expression^64^. Moreover, spliceosome-related pre-mRNA processing in neuronal dendrites may be associated with synapse function^37^, which has an important role in the development of depression^65^. In present results, spliceosome disruption was identified as the significant perturbed function in gut microbiota dysbiosis, characterized by the dysregulation of phosphorylation in U snRNA related proteins and complex components, such as SNRNP70, U2SURP, PRPF38A, HSPA8, RBMXL1, SRSF1, SRSF4, and SRSF7, WBP11 and HNRNPC in GF model, ACIN1, SLU7 and NCBP3 in FMT model. NCBP1 and TRA2B in both models. NCBP1 was also identified as the central protein in the PPI network analysis. This finding suggests that the NCBP1 phosphorylation may serve as a specific pathogenic target through which gut microbiota dysbiosis influences brain function and behavior. Importantly, NCBP1 is a component of the cap-binding complex (CBC) and has a pivotal role in various aspects of RNA biogenesis processes and the regulation of gene expression^63,66–68^ and is required for cell growth and viability. Associates with NCBP3 to form an alternative CBC, which has a key role in mRNA export and is particularly important in cellular stress situations such as virus infections. Previous findings have suggested that dysfunctional gene splicing was identified as a potential contributor to neuropsychiatric disorders (e.g., schizophrenia and affective disorders)^69^. These results support the conclusion that gut microbiota dysbiosis may disturb brain function through regulating RNA biogenesis processes and altering the associated gene expression, and the NCBP1 phosphorylation may serve as a key specific pathogenic target.

Some limitations of the present study need to be noted. First, only male mice were used to perform experiment and phosphoproteomic profiling, and the sex-specific effects of gut microbiota require further investigation. Second, only hippocampal phosphoprotein signatures were assessed. Thus, future studies should integrate the different brain regions and -omics data to enable a systematic analysis of the biological basis of host–microbial interplay. Third, further studies to validate the current findings are required. Finally, as previously described^6^, the use of pooled fecal samples from the subjects who were recruited from the same clinical site was regarded as a primary limitation. Further studies using individual fecal samples from ethnically diverse donors are required to identify the specific gut microbiotic strains that contribute to the development of depression.

## Conclusions

In summary, gut microbiota dysbiosis can cause mental disorders, such as depression, through the microbiota–gut–brain axis. In the present study, we demonstrated that the GF and FMT models exhibited global dysregulation in protein phosphorylation. These phosphorylation dysregulations were consistently associated with glutamatergic neurotransmitter system disturbances. And the FMT mice exhibited disturbances in lipid metabolism and amino acid metabolism in both the periphery and brain through integrating phosphoproteomic and metabolomic analysis. Moreover, CAMKII-CREB signaling pathway in neurons, in response to these disturbances, was the primary common perturbed cellular process. In addition, we demonstrated that the spliceosome, never directly implicated in mental disorders previously, was a significantly neuronal function disrupted by gut microbiota dysbiosis, and the NCBP1 phosphorylation was identified as a novel pathogenic target. These results present a new perspective to study the pathologic mechanisms of gut microbiota dysbiosis related depression and highlight potential gut-mediated therapies for depression.

## Supplementary information

Supplementary Materials
