# Supplementary material for: Integrated phosphoproteomic and metabolomic profiling reveals perturbed pathways in the hippocampus of gut microbiota dysbiosis mice
Source: Transl Psychiatry. 2020 Oct 13;10:346. doi: 10.1038/s41398-020-01024-9 (PMC7553953; doi:10.1038/s41398-020-01024-9)
Supplement: Supplementary file 1 — Supplementary Materials [file 41398_2020_1024_MOESM1_ESM.docx]

**Table S1.** Differential hippocampal phosphosites between germ-free (GF) and specific-pathogen-free (SPF) mice.

| **UniProtKB AC** | **Gene symbol** | **residues** | **Position** | **Fold Change (GF/SPF)** | **P-value** | **Regulated Type** |
| --- | --- | --- | --- | --- | --- | --- |
| A2A690 | Tanc2 | S | 1480 | 0.62 | 0.001 | Down |
| A2A690 | Tanc2 | S | 26 | 0.65 | 0.012 | Down |
| A2A690 | Tanc2 | S | 22 | 0.41 | 0.032 | Down |
| A2A9T0 | Tbkbp1 | S | 336 | 0.65 | 0.002 | Down |
| A2AAJ9 | Obscn | S | 8372 | 0.56 | 0.020 | Down |
| A2AH22 | Ambra1 | S | 1206 | 0.61 | 0.032 | Down |
| A2ALS5 | Rap1gap | S | 515 | 0.50 | 0.004 | Down |
| A2AN08 | Ubr4 | S | 1761 | 0.33 | 0.004 | Down |
| A2AP18 | Plch2 | S | 1012 | 0.34 | 0.009 | Down |
| B1AY10 | Nfx1 | S | 147 | 0.59 | 0.007 | Down |
| B1AY10 | Nfx1 | S | 149 | 0.56 | 0.015 | Down |
| B1AZA5 | Tmem245 | S | 12 | 0.51 | 0.035 | Down |
| D3YVF0 | Akap5 | S | 68 | 0.61 | 0.010 | Down |
| O08553 | Dpysl2 | S | 518 | 0.58 | 0.035 | Down |
| O08599 | Stxbp1 | S | 593 | 0.65 | 0.005 | Down |
| O08599 | Stxbp1 | S | 594 | 0.58 | 0.016 | Down |
| O35071 | Kif1c | S | 1031 | 0.46 | 0.005 | Down |
| O35668 | Hap1 | S | 615 | 0.28 | 0.010 | Down |
| O35681 | Syt3 | S | 586 | 0.63 | 0.024 | Down |
| O35927 | Ctnnd2 | S | 300 | 0.19 | 0.002 | Down |
| O35927 | Ctnnd2 | S | 296 | 0.19 | 0.002 | Down |
| O35927 | Ctnnd2 | S | 1094 | 0.64 | 0.005 | Down |
| O35927 | Ctnnd2 | S | 1098 | 0.59 | 0.026 | Down |
| O54991 | Cntnap1 | S | 1327 | 0.47 | 0.009 | Down |
| O70318 | Epb41l2 | S | 698 | 0.63 | 0.009 | Down |
| O88738 | Birc6 | S | 2245 | 0.39 | 0.004 | Down |
| O88746 | Tom1 | S | 180 | 0.53 | 0.001 | Down |
| O88935 | Syn1 | S | 427 | 0.52 | 0.016 | Down |
| P04370 | Mbp | T | 229 | 0.18 | 0.000 | Down |
| P04370 | Mbp | S | 245 | 0.41 | 0.010 | Down |
| P06837 | Gap43 | S | 144 | 0.01 | 0.000 | Down |
| P06837 | Gap43 | S | 142 | 0.18 | 0.001 | Down |
| P06837 | Gap43 | S | 128 | 0.63 | 0.009 | Down |
| P06837 | Gap43 | T | 95 | 0.63 | 0.014 | Down |
| P10637 | Mapt | S | 451 | 0.08 | 0.000 | Down |
| P11798 | Camk2a | T | 276 | 0.64 | 0.046 | Down |
| P14873 | Map1b | S | 2068 | 0.29 | 0.001 | Down |
| P14873 | Map1b | S | 1627 | 0.52 | 0.001 | Down |
| P14873 | Map1b | T | 1629 | 0.61 | 0.008 | Down |
| P16014 | Chgb | S | 229 | 0.23 | 0.001 | Down |
| P16014 | Chgb | S | 365 | 0.25 | 0.004 | Down |
| P16014 | Chgb | S | 234 | 0.42 | 0.005 | Down |
| P16014 | Chgb | S | 237 | 0.46 | 0.014 | Down |
| P16330 | Cnp | S | 22 | 0.28 | 0.002 | Down |
| P20357 | Map2 | S | 1013 | 0.34 | 0.005 | Down |
| P23475 | Xrcc6 | T | 290 | 0.39 | 0.008 | Down |
| P26645 | Marcks | S | 163 | 0.66 | 0.022 | Down |
| P28667 | Marcksl1 | S | 104 | 0.62 | 0.010 | Down |
| P28667 | Marcksl1 | T | 148 | 0.64 | 0.016 | Down |
| P35436 | Grin2a | S | 1198 | 0.48 | 0.004 | Down |
| P35438 | Grin1 | S | 897 | 0.26 | 0.001 | Down |
| P39053 | Dnm1 | S | 774 | 0.47 | 0.001 | Down |
| P39053 | Dnm1 | S | 778 | 0.30 | 0.003 | Down |
| P39053 | Dnm1 | T | 776 | 0.52 | 0.010 | Down |
| P39447 | Tjp1 | S | 315 | 0.39 | 0.006 | Down |
| P39447 | Tjp1 | S | 313 | 0.39 | 0.006 | Down |
| P39447 | Tjp1 | S | 311 | 0.39 | 0.006 | Down |
| P47708 | Rph3a | S | 271 | 0.29 | 0.003 | Down |
| P47746 | Cnr1 | S | 442 | 0.60 | 0.002 | Down |
| P47746 | Cnr1 | T | 440 | 0.61 | 0.039 | Down |
| P48318 | Gad1 | S | 55 | 0.61 | 0.011 | Down |
| P51830 | Adcy9 | S | 365 | 0.08 | 0.000 | Down |
| P54731 | Faf1 | S | 269 | 0.42 | 0.021 | Down |
| P56399 | Usp5 | S | 751 | 0.48 | 0.040 | Down |
| P56564 | Slc1a3 | S | 4 | 0.57 | 0.023 | Down |
| P56564 | Slc1a3 | T | 2 | 0.57 | 0.023 | Down |
| P59281 | Arhgap39 | S | 123 | 0.32 | 0.002 | Down |
| P59648 | Fxyd7 | S | 60 | 0.36 | 0.000 | Down |
| P59648 | Fxyd7 | S | 56 | 0.40 | 0.001 | Down |
| P60879 | Snap25 | T | 138 | 0.42 | 0.008 | Down |
| P61982 | Ywhag | S | 71 | 0.36 | 0.002 | Down |
| P63017 | Hspa8 | S | 254 | 0.57 | 0.041 | Down |
| P63094 | Gnas | S | 352 | 0.49 | 0.017 | Down |
| P70255 | Nfic | S | 194 | 0.60 | 0.011 | Down |
| P70336 | Rock2 | S | 1052 | 0.42 | 0.001 | Down |
| P97412 | Lyst | S | 2564 | 0.65 | 0.004 | Down |
| P97412 | Lyst | S | 2560 | 0.64 | 0.030 | Down |
| P97433 | Arhgef28 | S | 1198 | 0.45 | 0.005 | Down |
| P97445 | Cacna1a | S | 2329 | 0.45 | 0.000 | Down |
| Q00PI9 | Hnrnpul2 | S | 191 | 0.41 | 0.023 | Down |
| Q04735 | Cdk16 | S | 12 | 0.60 | 0.034 | Down |
| Q05512 | Mark2 | S | 400 | 0.50 | 0.006 | Down |
| Q05D44 | Eif5b | S | 165 | 0.59 | 0.010 | Down |
| Q08460 | Kcnma1 | S | 728 | 0.46 | 0.006 | Down |
| Q08460 | Kcnma1 | S | 724 | 0.61 | 0.027 | Down |
| Q0PHV7 | Dact3 | S | 268 | 0.55 | 0.001 | Down |
| Q148V7 | Kiaa1468 | S | 193 | 0.45 | 0.002 | Down |
| Q3TLH4 | Prrc2c | S | 899 | 0.60 | 0.013 | Down |
| Q3TQI7 | C9orf78 | S | 15 | 0.56 | 0.041 | Down |
| Q3TQI7 | C9orf78 | S | 17 | 0.53 | 0.048 | Down |
| Q3U1Y4 | Dennd4b | S | 735 | 0.63 | 0.004 | Down |
| Q3U2I3 | Fam160a2 | T | 876 | 0.32 | 0.018 | Down |
| Q3U2I3 | Fam160a2 | S | 874 | 0.32 | 0.018 | Down |
| Q3UHB8 | Ccdc177 | S | 423 | 0.27 | 0.002 | Down |
| Q3UHD9 | Agap2 | S | 650 | 0.15 | 0.004 | Down |
| Q3UHD9 | Agap2 | S | 642 | 0.58 | 0.007 | Down |
| Q3UHD9 | Agap2 | T | 803 | 0.60 | 0.022 | Down |
| Q3UHE1 | Pitpnm3 | S | 31 | 0.37 | 0.040 | Down |
| Q3UHE1 | Pitpnm3 | S | 30 | 0.37 | 0.040 | Down |
| Q3UHE1 | Pitpnm3 | S | 27 | 0.37 | 0.040 | Down |
| Q3UL36 | Arglu1 | S | 75 | 0.29 | 0.004 | Down |
| Q3UNH4 | Gprin1 | S | 622 | 0.59 | 0.008 | Down |
| Q3UNH4 | Gprin1 | S | 620 | 0.55 | 0.016 | Down |
| Q3USH5 | Sfswap | S | 812 | 0.30 | 0.002 | Down |
| Q3UVX5 | Grm5 | S | 851 | 0.35 | 0.004 | Down |
| Q3UYV9 | Ncbp1 | S | 674 | 0.05 | 0.004 | Down |
| Q4ACU6 | Shank3 | S | 781 | 0.60 | 0.027 | Down |
| Q4KUS2 | Unc13a | T | 416 | 0.27 | 0.002 | Down |
| Q52KI8 | Srrm1 | T | 414 | 0.48 | 0.013 | Down |
| Q5GH67 | Xkr4 | S | 214 | 0.09 | 0.000 | Down |
| Q5SP85 | Ccdc85a | S | 240 | 0.40 | 0.002 | Down |
| Q5U3K5 | Rabl6 | S | 638 | 0.44 | 0.003 | Down |
| Q60610 | Tiam1 | S | 1437 | 0.50 | 0.008 | Down |
| Q60668 | Hnrnpd | S | 80 | 0.57 | 0.013 | Down |
| Q60668 | Hnrnpd | S | 83 | 0.63 | 0.021 | Down |
| Q60675 | Lama2 | S | 27 | 0.26 | 0.027 | Down |
| Q60875 | Arhgef2 | S | 718 | 0.28 | 0.001 | Down |
| Q61136 | Prpf4b | S | 233 | 0.06 | 0.000 | Down |
| Q61136 | Prpf4b | S | 231 | 0.06 | 0.000 | Down |
| Q61234 | Snta1 | S | 194 | 0.66 | 0.017 | Down |
| Q61337 | Bad | S | 170 | 0.63 | 0.013 | Down |
| Q61789 | Lama3 | Y | 1670 | 0.66 | 0.035 | Down |
| Q61789 | Lama3 | Y | 1669 | 0.66 | 0.035 | Down |
| Q62188 | Dpysl3 | T | 514 | 0.40 | 0.001 | Down |
| Q62261 | Sptbn1 | T | 2316 | 0.42 | 0.005 | Down |
| Q62261 | Sptbn1 | S | 2318 | 0.64 | 0.010 | Down |
| Q62261 | Sptbn1 | T | 2319 | 0.57 | 0.015 | Down |
| Q62277 | Syp | T | 190 | 0.11 | 0.000 | Down |
| Q62376 | Snrnp70 | S | 268 | 0.21 | 0.001 | Down |
| Q62407 | Speg | S | 2114 | 0.60 | 0.002 | Down |
| Q62414 | Neurod2 | S | 317 | 0.28 | 0.001 | Down |
| Q62433 | Ndrg1 | T | 346 | 0.36 | 0.009 | Down |
| Q62441 | Tle4 | S | 222 | 0.61 | 0.010 | Down |
| Q640R3 | Hepacam | S | 386 | 0.63 | 0.005 | Down |
| Q64707 | Zrsr1 | S | 418 | 0.60 | 0.021 | Down |
| Q64707 | Zrsr1 | S | 417 | 0.57 | 0.027 | Down |
| Q66JV4 | Rbm12b2 | S | 254 | 0.44 | 0.006 | Down |
| Q66L44 | Cbarp | S | 591 | 0.39 | 0.001 | Down |
| Q66L44 | Cbarp | S | 320 | 0.57 | 0.048 | Down |
| Q69Z98 | Brsk2 | S | 436 | 0.63 | 0.003 | Down |
| Q69ZW3 | Ehbp1 | S | 1038 | 0.60 | 0.024 | Down |
| Q6A0A2 | Larp4b | S | 721 | 0.62 | 0.049 | Down |
| Q6DFV7 | Ncoa7 | S | 353 | 0.62 | 0.000 | Down |
| Q6I6G8 | Hecw2 | S | 1048 | 0.65 | 0.036 | Down |
| Q6NS60 | Fbxo41 | S | 396 | 0.34 | 0.000 | Down |
| Q6NS60 | Fbxo41 | S | 395 | 0.46 | 0.011 | Down |
| Q6NV83 | U2surp | S | 932 | 0.45 | 0.007 | Down |
| Q6NV83 | U2surp | S | 930 | 0.45 | 0.007 | Down |
| Q6NZJ6 | Eif4g1 | S | 1597 | 0.20 | 0.007 | Down |
| Q6P9R1 | Ddx51 | S | 77 | 0.53 | 0.047 | Down |
| Q6PAJ1 | Bcr | S | 303 | 0.31 | 0.011 | Down |
| Q6PAR5 | Gapvd1 | S | 964 | 0.22 | 0.002 | Down |
| Q6PCN3 | Ttbk1 | S | 655 | 0.40 | 0.002 | Down |
| Q6PCN3 | Ttbk1 | S | 456 | 0.60 | 0.041 | Down |
| Q6PDM2 | Srsf1 | S | 201 | 0.59 | 0.032 | Down |
| Q6PDM2 | Srsf1 | S | 199 | 0.59 | 0.032 | Down |
| Q6PER3 | Mapre3 | T | 161 | 0.25 | 0.002 | Down |
| Q6PER3 | Mapre3 | S | 162 | 0.26 | 0.003 | Down |
| Q6PFD5 | Dlgap3 | S | 429 | 0.23 | 0.000 | Down |
| Q6PFR5 | Tra2a | S | 73 | 0.18 | 0.001 | Down |
| Q6PFR5 | Tra2a | S | 71 | 0.18 | 0.001 | Down |
| Q6PKN7 | Inca1 | S | 178 | 0.22 | 0.018 | Down |
| Q6PKN7 | Inca1 | Y | 177 | 0.22 | 0.018 | Down |
| Q6ZPZ3 | Zc3h4 | S | 94 | 0.25 | 0.000 | Down |
| Q6ZPZ3 | Zc3h4 | S | 92 | 0.25 | 0.000 | Down |
| Q6ZQ08 | Cnot1 | S | 1480 | 0.28 | 0.002 | Down |
| Q6ZQ18 | Efr3b | S | 709 | 0.55 | 0.012 | Down |
| Q6ZQ58 | Larp1 | S | 494 | 0.62 | 0.016 | Down |
| Q76LS9 | Mindy1 | S | 382 | 0.57 | 0.005 | Down |
| Q7TME0 | Plppr4 | S | 746 | 0.24 | 0.005 | Down |
| Q7TSJ2 | Map6 | S | 198 | 0.61 | 0.001 | Down |
| Q7TT50 | Cdc42bpb | S | 1692 | 0.58 | 0.012 | Down |
| Q7TT50 | Cdc42bpb | S | 1695 | 0.59 | 0.015 | Down |
| Q7TT50 | Cdc42bpb | S | 481 | 0.65 | 0.032 | Down |
| Q80TI0 | Gramd1b | S | 601 | 0.52 | 0.008 | Down |
| Q80TL7 | Mon2 | S | 1213 | 0.62 | 0.001 | Down |
| Q80TV8 | Clasp1 | S | 572 | 0.43 | 0.003 | Down |
| Q80U28 | Madd | S | 929 | 0.58 | 0.025 | Down |
| Q80XU3 | Nucks1 | S | 19 | 0.66 | 0.021 | Down |
| Q80XU8 | Lrfn4 | S | 627 | 0.51 | 0.023 | Down |
| Q80Y81 | Elac2 | S | 191 | 0.44 | 0.000 | Down |
| Q80YN3 | Bcas1 | S | 601 | 0.65 | 0.002 | Down |
| Q812A2 | Srgap3 | S | 948 | 0.62 | 0.009 | Down |
| Q8BGB5 | Limd2 | S | 32 | 0.62 | 0.001 | Down |
| Q8BGD9 | Eif4b | S | 422 | 0.61 | 0.022 | Down |
| Q8BI72 | Cdkn2aip | S | 169 | 0.35 | 0.001 | Down |
| Q8BL97 | Srsf7 | S | 262 | 0.64 | 0.019 | Down |
| Q8BL97 | Srsf7 | S | 260 | 0.64 | 0.019 | Down |
| Q8BLN6 | Unc80 | S | 166 | 0.42 | 0.004 | Down |
| Q8BLN6 | Unc80 | S | 6 | 0.48 | 0.016 | Down |
| Q8BLR2 | Cpne4 | T | 137 | 0.25 | 0.002 | Down |
| Q8BPN8 | Dmxl2 | S | 473 | 0.24 | 0.001 | Down |
| Q8BQP9 | Rgs7bp | S | 38 | 0.11 | 0.000 | Down |
| Q8BQP9 | Rgs7bp | S | 40 | 0.15 | 0.000 | Down |
| Q8BRT1 | Clasp2 | S | 376 | 0.62 | 0.009 | Down |
| Q8BRT1 | Clasp2 | S | 547 | 0.47 | 0.024 | Down |
| Q8BRT1 | Clasp2 | S | 542 | 0.47 | 0.024 | Down |
| Q8BRT1 | Clasp2 | S | 374 | 0.62 | 0.025 | Down |
| Q8BTG3 | Tcp11l1 | S | 44 | 0.14 | 0.003 | Down |
| Q8BTG3 | Tcp11l1 | S | 55 | 0.63 | 0.028 | Down |
| Q8BTI8 | Srrm2 | S | 1825 | 0.50 | 0.000 | Down |
| Q8BTI8 | Srrm2 | S | 1822 | 0.30 | 0.001 | Down |
| Q8BTI8 | Srrm2 | S | 2656 | 0.52 | 0.004 | Down |
| Q8BTI8 | Srrm2 | Y | 1776 | 0.57 | 0.018 | Down |
| Q8BTI8 | Srrm2 | T | 877 | 0.64 | 0.028 | Down |
| Q8BTI8 | Srrm2 | S | 882 | 0.65 | 0.035 | Down |
| Q8BTV2 | Cpsf7 | S | 194 | 0.48 | 0.023 | Down |
| Q8BTV2 | Cpsf7 | S | 197 | 0.30 | 0.025 | Down |
| Q8BVF2 | Pdcl3 | S | 235 | 0.58 | 0.005 | Down |
| Q8BVL3 | Snx17 | S | 409 | 0.34 | 0.002 | Down |
| Q8BX57 | Pxk | S | 448 | 0.46 | 0.006 | Down |
| Q8BX57 | Pxk | S | 452 | 0.43 | 0.021 | Down |
| Q8BX70 | Vps13c | S | 841 | 0.61 | 0.012 | Down |
| Q8BXK8 | Agap1 | S | 339 | 0.65 | 0.013 | Down |
| Q8BYM5 | Nlgn3 | S | 722 | 0.56 | 0.011 | Down |
| Q8BZX4 | Srek1 | S | 465 | 0.34 | 0.004 | Down |
| Q8C129 | Lnpep | S | 91 | 0.52 | 0.023 | Down |
| Q8C1W1 | Vash1 | S | 341 | 0.17 | 0.001 | Down |
| Q8C419 | Gpr158 | S | 239 | 0.62 | 0.008 | Down |
| Q8C561 | Lmbrd2 | S | 610 | 0.40 | 0.005 | Down |
| Q8C8R3 | Ank2 | S | 3411 | 0.36 | 0.002 | Down |
| Q8C8R3 | Ank2 | S | 1844 | 0.61 | 0.011 | Down |
| Q8C8R3 | Ank2 | S | 1842 | 0.45 | 0.018 | Down |
| Q8C8T7 | Elfn1 | S | 629 | 0.66 | 0.012 | Down |
| Q8CBE3 | Wdr37 | S | 30 | 0.12 | 0.001 | Down |
| Q8CBE3 | Wdr37 | T | 28 | 0.07 | 0.002 | Down |
| Q8CBW3 | Abi1 | S | 243 | 0.31 | 0.001 | Down |
| Q8CBW3 | Abi1 | S | 296 | 0.41 | 0.024 | Down |
| Q8CBW3 | Abi1 | T | 297 | 0.35 | 0.032 | Down |
| Q8CBW3 | Abi1 | S | 222 | 0.33 | 0.047 | Down |
| Q8CCJ3 | Ufl1 | S | 458 | 0.58 | 0.026 | Down |
| Q8CD78 | Sertm1 | S | 106 | 0.63 | 0.004 | Down |
| Q8CHX7 | Rftn2 | S | 189 | 0.52 | 0.016 | Down |
| Q8JZP2 | Syn3 | S | 519 | 0.48 | 0.005 | Down |
| Q8JZP9 | Gas2l1 | S | 352 | 0.56 | 0.008 | Down |
| Q8K212 | Pacs1 | S | 526 | 0.13 | 0.002 | Down |
| Q8K3M5 | Cables2 | S | 150 | 0.01 | 0.000 | Down |
| Q8N7N5 | Dcaf8 | S | 124 | 0.54 | 0.006 | Down |
| Q8N7N5 | Dcaf8 | S | 123 | 0.54 | 0.006 | Down |
| Q8R001 | Mapre2 | S | 199 | 0.60 | 0.015 | Down |
| Q8R0L9 | Tada3 | T | 297 | 0.52 | 0.022 | Down |
| Q8R0S4 | Cacnb4 | S | 507 | 0.56 | 0.008 | Down |
| Q8R149 | Bud13 | T | 262 | 0.52 | 0.011 | Down |
| Q8R149 | Bud13 | S | 272 | 0.31 | 0.012 | Down |
| Q8R3Y8 | Irf2bp1 | S | 453 | 0.63 | 0.000 | Down |
| Q8VCY8 | Plppr2 | S | 330 | 0.59 | 0.019 | Down |
| Q8VDJ3 | Hdlbp | S | 944 | 0.65 | 0.043 | Down |
| Q8VDN4 | Ccdc92 | S | 261 | 0.48 | 0.007 | Down |
| Q8VE28 | Nkd2 | S | 296 | 0.60 | 0.030 | Down |
| Q8VE97 | Srsf4 | S | 289 | 0.04 | 0.000 | Down |
| Q8VE97 | Srsf4 | S | 291 | 0.04 | 0.000 | Down |
| Q8VHW2 | Cacng8 | S | 237 | 0.09 | 0.000 | Down |
| Q8VHW2 | Cacng8 | S | 280 | 0.30 | 0.003 | Down |
| Q8VHW2 | Cacng8 | S | 277 | 0.34 | 0.005 | Down |
| Q91V14 | Slc12a5 | S | 27 | 0.32 | 0.002 | Down |
| Q91VM5 | Rbmxl1 | T | 163 | 0.63 | 0.046 | Down |
| Q91W39 | Ncoa5 | S | 34 | 0.65 | 0.050 | Down |
| Q91Z67 | Srgap2 | S | 206 | 0.62 | 0.049 | Down |
| Q921Q7 | Rin1 | S | 280 | 0.66 | 0.029 | Down |
| Q99J21 | Mcoln1 | S | 10 | 0.49 | 0.018 | Down |
| Q99M08 | C4orf3 | S | 19 | 0.35 | 0.000 | Down |
| Q99MJ9 | Ddx50 | S | 41 | 0.59 | 0.004 | Down |
| Q99MJ9 | Ddx50 | S | 39 | 0.35 | 0.006 | Down |
| Q99N57 | Raf1 | S | 43 | 0.38 | 0.020 | Down |
| Q99NE5 | Rims1 | S | 1240 | 0.62 | 0.009 | Down |
| Q9CQU1 | Mfap1 | S | 133 | 0.46 | 0.001 | Down |
| Q9CQU1 | Mfap1 | S | 132 | 0.46 | 0.001 | Down |
| Q9CRA5 | Golph3 | S | 35 | 0.53 | 0.002 | Down |
| Q9CXW2 | Mrps22 | S | 184 | 0.22 | 0.004 | Down |
| Q9D2P8 | Mobp | S | 74 | 0.58 | 0.006 | Down |
| Q9D2P8 | Mobp | T | 73 | 0.55 | 0.030 | Down |
| Q9D4F2 | Plpp6 | S | 60 | 0.63 | 0.004 | Down |
| Q9D6E4 | Ttc9b | S | 226 | 0.54 | 0.007 | Down |
| Q9D898 | Arpc5l | S | 64 | 0.55 | 0.010 | Down |
| Q9DBH0 | Wwp2 | S | 211 | 0.65 | 0.038 | Down |
| Q9EPQ8 | Tcf20 | S | 1819 | 0.60 | 0.002 | Down |
| Q9EQH3 | Vps35 | S | 203 | 0.08 | 0.009 | Down |
| Q9ESN9 | Mapk8ip3 | S | 755 | 0.65 | 0.001 | Down |
| Q9ESN9 | Mapk8ip3 | S | 564 | 0.50 | 0.006 | Down |
| Q9EST5 | Anp32b | T | 265 | 0.55 | 0.007 | Down |
| Q9ET80 | Jph1 | S | 490 | 0.51 | 0.033 | Down |
| Q9JK45 | Kcnq5 | S | 442 | 0.50 | 0.008 | Down |
| Q9QYB8 | Add2 | S | 618 | 0.53 | 0.013 | Down |
| Q9QYB8 | Add2 | S | 614 | 0.61 | 0.018 | Down |
| Q9QYC0 | Add1 | S | 465 | 0.56 | 0.009 | Down |
| Q9QYR6 | Map1a | S | 1634 | 0.65 | 0.011 | Down |
| Q9QYR6 | Map1a | T | 1633 | 0.57 | 0.028 | Down |
| Q9QYR6 | Map1a | S | 611 | 0.64 | 0.042 | Down |
| Q9QYX7 | Pclo | T | 1390 | 0.56 | 0.023 | Down |
| Q9R020 | Zranb2 | S | 316 | 0.02 | 0.000 | Down |
| Q9R078 | Prkab1 | S | 24 | 0.01 | 0.000 | Down |
| Q9WUB4 | Dctn6 | T | 186 | 0.34 | 0.005 | Down |
| Q9WV18 | Gabbr1 | S | 878 | 0.45 | 0.010 | Down |
| Q9WVQ1 | Magi2 | S | 884 | 0.61 | 0.005 | Down |
| Q9WVQ1 | Magi2 | S | 883 | 0.61 | 0.009 | Down |
| Q9Z1B3 | Plcb1 | S | 477 | 0.02 | 0.000 | Down |
| Q9Z1R2 | Bag6 | T | 985 | 0.62 | 0.000 | Down |
| Q9Z2E9 | Bscl2 | S | 280 | 0.34 | 0.017 | Down |
| Q9Z351 | Kcnq2 | S | 429 | 0.64 | 0.009 | Down |
| O35691 | Pnn | S | 100 | 1.63 | 0.016 | Up |
| O55106 | Strn | S | 245 | 1.57 | 0.043 | Up |
| O89032 | Sh3pxd2a | S | 993 | 1.68 | 0.044 | Up |
| P05064 | Aldoa | S | 39 | 1.51 | 0.023 | Up |
| P08553 | Nefm | S | 545 | 2.34 | 0.043 | Up |
| P14873 | Map1b | S | 989 | 1.70 | 0.049 | Up |
| P16054 | Prkce | T | 710 | 3.40 | 0.004 | Up |
| P16546 | Sptan1 | S | 1031 | 1.63 | 0.015 | Up |
| P23242 | Gja1 | S | 373 | 2.57 | 0.017 | Up |
| P23242 | Gja1 | S | 364 | 1.95 | 0.023 | Up |
| P23242 | Gja1 | S | 372 | 2.20 | 0.035 | Up |
| P58871 | Tnks1bp1 | S | 178 | 1.64 | 0.029 | Up |
| P62996 | Tra2b | S | 22 | 2.42 | 0.039 | Up |
| P62996 | Tra2b | S | 20 | 2.42 | 0.039 | Up |
| Q00PI9 | Hnrnpul2 | S | 159 | 1.50 | 0.016 | Up |
| Q05512 | Mark2 | T | 208 | 1.55 | 0.005 | Up |
| Q3UH99 | Shisa6 | S | 416 | 1.90 | 0.004 | Up |
| Q3UHL1 | Camkv | S | 392 | 1.52 | 0.028 | Up |
| Q68EF6 | Begain | S | 570 | 1.69 | 0.015 | Up |
| Q6R891 | Ppp1r9b | S | 192 | 1.76 | 0.049 | Up |
| Q80ZF8 | Adgrb3 | S | 1220 | 1.68 | 0.049 | Up |
| Q812A2 | Srgap3 | S | 954 | 1.53 | 0.030 | Up |
| Q8C0T5 | Sipa1l1 | S | 258 | 1.56 | 0.048 | Up |
| Q8K3X4 | Irf2bpl | S | 195 | 1.55 | 0.014 | Up |
| Q91WM6 | Eva1a | S | 118 | 1.89 | 0.007 | Up |
| Q9D394 | Rufy3 | S | 34 | 1.73 | 0.004 | Up |
| Q9DBR0 | Akap8 | S | 336 | 1.50 | 0.006 | Up |
| Q9WTU3 | Scn8a | S | 600 | 1.70 | 0.007 | Up |

**Table S2.** Differential hippocampal phosphosites between FMT-MDD and FMT-HC mice.

| **UniProtKB AC** | **Gene symbol** | **residues** | **Position** | **Fold Change (MDD/HC)** | **P-value** | **Regulated Type** |
| --- | --- | --- | --- | --- | --- | --- |
| A2A7S8 | Kiaa1522 | S | 543 | 0.27 | 0.020 | Down |
| A2A7S8 | Kiaa1522 | S | 402 | 0.46 | 0.007 | Down |
| A2A7S8 | Kiaa1522 | S | 909 | 0.54 | 0.005 | Down |
| A2AFR3 | Frmpd4 | S | 1004 | 0.10 | 0.006 | Down |
| A2AJA9 | Gm996 | S | 397 | 0.58 | 0.030 | Down |
| E9PUL5 | Prrt2 | S | 100 | 0.66 | 0.016 | Down |
| E9Q1P8 | Irf2bp2 | S | 169 | 0.51 | 0.001 | Down |
| F6ZDS4 | Tpr | S | 2141 | 0.61 | 0.003 | Down |
| G3X939 | Slc9a3 | S | 550 | 0.42 | 0.006 | Down |
| O08638 | Myh11 | S | 8 | 0.16 | 0.004 | Down |
| O08638 | Myh11 | S | 1954 | 0.39 | 0.001 | Down |
| O08796 | Eef2k | S | 473 | 0.60 | 0.024 | Down |
| O08796 | Eef2k | S | 469 | 0.63 | 0.001 | Down |
| O09044 | Snap23 | S | 110 | 0.51 | 0.007 | Down |
| O35691 | Pnn | S | 68 | 0.50 | 0.010 | Down |
| O54724 | Ptrf | S | 42 | 0.30 | 0.000 | Down |
| O54724 | Ptrf | T | 40 | 0.43 | 0.001 | Down |
| O54786 | Dffa | S | 314 | 0.50 | 0.018 | Down |
| O54825 | Bysl | S | 97 | 0.54 | 0.002 | Down |
| O54916 | Reps1 | S | 539 | 0.47 | 0.008 | Down |
| O54916 | Reps1 | S | 708 | 0.58 | 0.004 | Down |
| O55098 | Stk10 | T | 949 | 0.37 | 0.000 | Down |
| O55112 | Aff2 | S | 580 | 0.14 | 0.002 | Down |
| O55112 | Aff2 | T | 581 | 0.14 | 0.002 | Down |
| O70475 | Ugdh | T | 474 | 0.22 | 0.000 | Down |
| O70585 | Dtnb | S | 528 | 0.27 | 0.021 | Down |
| O70585 | Dtnb | T | 527 | 0.59 | 0.002 | Down |
| O88343 | Slc4a4 | S | 257 | 0.39 | 0.001 | Down |
| O88874 | Ccnk | S | 341 | 0.65 | 0.025 | Down |
| O89110 | Casp8 | S | 188 | 0.28 | 0.000 | Down |
| P00405 | Mtco2 | Y | 218 | 0.44 | 0.001 | Down |
| P06537 | Nr3c1 | S | 275 | 0.50 | 0.001 | Down |
| P06745 | Gpi | S | 455 | 0.64 | 0.003 | Down |
| P08122 | Col4a2 | S | 1470 | 0.66 | 0.013 | Down |
| P08775 | Polr2a | S | 1861 | 0.58 | 0.042 | Down |
| P09470 | Ace | S | 1305 | 0.38 | 0.002 | Down |
| P11679 | Krt8 | S | 24 | 0.29 | 0.004 | Down |
| P11679 | Krt8 | S | 489 | 0.38 | 0.019 | Down |
| P14602 | Hspb1 | S | 13 | 0.29 | 0.001 | Down |
| P17095 | Hmga1 | S | 36 | 0.65 | 0.001 | Down |
| P19973 | Lsp1 | S | 243 | 0.62 | 0.001 | Down |
| P20152 | Vim | S | 325 | 0.64 | 0.047 | Down |
| P20357 | Map2 | S | 1487 | 0.66 | 0.004 | Down |
| P21447 | Abcb1a | S | 667 | 0.51 | 0.004 | Down |
| P24788 | Cdk11b | S | 578 | 0.53 | 0.007 | Down |
| P26231 | Ctnna1 | S | 641 | 0.59 | 0.001 | Down |
| P27546 | Map4 | S | 798 | 0.20 | 0.000 | Down |
| P27546 | Map4 | S | 475 | 0.55 | 0.026 | Down |
| P28867 | Prkcd | S | 504 | 0.65 | 0.021 | Down |
| P29699 | Ahsg | S | 134 | 0.49 | 0.022 | Down |
| P29699 | Ahsg | S | 138 | 0.60 | 0.001 | Down |
| P30999 | Ctnnd1 | T | 916 | 0.23 | 0.004 | Down |
| P30999 | Ctnnd1 | S | 349 | 0.26 | 0.000 | Down |
| P30999 | Ctnnd1 | S | 920 | 0.42 | 0.009 | Down |
| P31001 | Des | S | 28 | 0.23 | 0.001 | Down |
| P31001 | Des | S | 31 | 0.41 | 0.005 | Down |
| P31001 | Des | S | 32 | 0.66 | 0.002 | Down |
| P39447 | Tjp1 | S | 912 | 0.45 | 0.001 | Down |
| P39447 | Tjp1 | S | 277 | 0.57 | 0.010 | Down |
| P39447 | Tjp1 | S | 280 | 0.57 | 0.010 | Down |
| P43276 | Hist1h1b | S | 18 | 0.12 | 0.000 | Down |
| P46467 | Vps4b | S | 102 | 0.56 | 0.003 | Down |
| P46938 | Yap1 | T | 95 | 0.52 | 0.002 | Down |
| P47212 | Gal | S | 117 | 0.35 | 0.004 | Down |
| P47856 | Gfpt1 | S | 259 | 0.27 | 0.006 | Down |
| P49586 | Pcyt1a | S | 323 | 0.50 | 0.005 | Down |
| P49586 | Pcyt1a | S | 319 | 0.61 | 0.015 | Down |
| P51432 | Plcb3 | S | 480 | 0.07 | 0.001 | Down |
| P51432 | Plcb3 | S | 474 | 0.22 | 0.001 | Down |
| P51432 | Plcb3 | S | 537 | 0.26 | 0.009 | Down |
| P54276 | Msh6 | S | 62 | 0.61 | 0.001 | Down |
| P57016 | Lad1 | S | 259 | 0.03 | 0.001 | Down |
| P57016 | Lad1 | S | 62 | 0.26 | 0.000 | Down |
| P57016 | Lad1 | S | 523 | 0.46 | 0.000 | Down |
| P57016 | Lad1 | S | 328 | 0.53 | 0.002 | Down |
| P58501 | Paxbp1 | S | 16 | 0.61 | 0.002 | Down |
| P58871 | Tnks1bp1 | S | 1657 | 0.52 | 0.001 | Down |
| P59242 | Cgn | S | 445 | 0.22 | 0.003 | Down |
| P59242 | Cgn | S | 90 | 0.65 | 0.000 | Down |
| P59648 | Fxyd7 | S | 72 | 0.55 | 0.032 | Down |
| P59997 | Kdm2a | S | 739 | 0.50 | 0.004 | Down |
| P59997 | Kdm2a | S | 740 | 0.50 | 0.004 | Down |
| P62242 | Rps8 | T | 130 | 0.62 | 0.001 | Down |
| P62806 | Hist1h4a | S | 48 | 0.60 | 0.010 | Down |
| P62908 | Rps3 | T | 221 | 0.65 | 0.019 | Down |
| P62996 | Tra2b | T | 33 | 0.60 | 0.046 | Down |
| P63141 | Kcna2 | S | 468 | 0.46 | 0.001 | Down |
| P70255 | Nfic | S | 323 | 0.44 | 0.001 | Down |
| P70336 | Rock2 | S | 549 | 0.49 | 0.011 | Down |
| P70336 | Rock2 | S | 550 | 0.49 | 0.011 | Down |
| P70336 | Rock2 | S | 553 | 0.49 | 0.011 | Down |
| P70336 | Rock2 | T | 554 | 0.49 | 0.011 | Down |
| P70441 | Slc9a3r1 | S | 286 | 0.33 | 0.006 | Down |
| P70441 | Slc9a3r1 | S | 285 | 0.43 | 0.000 | Down |
| P70441 | Slc9a3r1 | S | 283 | 0.51 | 0.001 | Down |
| P70441 | Slc9a3r1 | S | 289 | 0.58 | 0.004 | Down |
| P83510 | Tnik | S | 651 | 0.51 | 0.003 | Down |
| P97360 | Etv6 | S | 215 | 0.45 | 0.000 | Down |
| P97496 | Smarcc1 | S | 327 | 0.54 | 0.001 | Down |
| P97496 | Smarcc1 | S | 329 | 0.54 | 0.001 | Down |
| P97496 | Smarcc1 | S | 309 | 0.55 | 0.002 | Down |
| Q01097 | Grin2b | S | 1477 | 0.38 | 0.049 | Down |
| Q01320 | Top2a | S | 1521 | 0.30 | 0.003 | Down |
| Q02111 | Prkcq | S | 212 | 0.61 | 0.048 | Down |
| Q02248 | Ctnnb1 | S | 191 | 0.48 | 0.016 | Down |
| Q03265 | Atp5a1 | S | 76 | 0.66 | 0.015 | Down |
| Q0VG18 | Smim24 | S | 109 | 0.25 | 0.003 | Down |
| Q0VG18 | Smim24 | S | 110 | 0.27 | 0.000 | Down |
| Q148V8 | Fam83h | S | 970 | 0.23 | 0.005 | Down |
| Q148V8 | Fam83h | S | 871 | 0.25 | 0.001 | Down |
| Q148V8 | Fam83h | S | 948 | 0.32 | 0.006 | Down |
| Q148V8 | Fam83h | S | 926 | 0.61 | 0.000 | Down |
| Q2TBA3 | Malt1 | S | 2 | 0.10 | 0.001 | Down |
| Q2TBA3 | Malt1 | S | 11 | 0.10 | 0.001 | Down |
| Q32NZ6 | Tmc5 | S | 291 | 0.52 | 0.011 | Down |
| Q3TJD7 | Pdlim7 | T | 96 | 0.45 | 0.001 | Down |
| Q3TLH4 | Prrc2c | S | 899 | 0.62 | 0.027 | Down |
| Q3U9G9 | Lbr | S | 103 | 0.45 | 0.000 | Down |
| Q3UH06 | Rreb1 | S | 175 | 0.35 | 0.006 | Down |
| Q3UHF7 | Hivep2 | S | 420 | 0.30 | 0.004 | Down |
| Q3UHJ0 | Aak1 | T | 638 | 0.65 | 0.033 | Down |
| Q3UJV1 | Ccdc61 | S | 330 | 0.59 | 0.039 | Down |
| Q3UJV1 | Ccdc61 | S | 332 | 0.59 | 0.039 | Down |
| Q3UQ44 | Iqgap2 | S | 16 | 0.52 | 0.034 | Down |
| Q3UQN2 | Fcho2 | S | 394 | 0.51 | 0.008 | Down |
| Q3UQU0 | Brd9 | S | 565 | 0.62 | 0.011 | Down |
| Q3UTJ2 | Sorbs2 | S | 339 | 0.62 | 0.002 | Down |
| Q3UY34 | unknown | S | 139 | 0.21 | 0.003 | Down |
| Q3UYV9 | Ncbp1 | S | 7 | 0.55 | 0.012 | Down |
| Q497N7 | unknown | S | 83 | 0.58 | 0.019 | Down |
| Q4ACU6 | Shank3 | S | 482 | 0.66 | 0.013 | Down |
| Q4V9W2 | Srek1ip1 | S | 143 | 0.57 | 0.001 | Down |
| Q4V9W2 | Srek1ip1 | S | 94 | 0.58 | 0.000 | Down |
| Q501J7 | Phactr4 | S | 457 | 0.36 | 0.021 | Down |
| Q52KI8 | Srrm1 | S | 743 | 0.62 | 0.009 | Down |
| Q52KI8 | Srrm1 | T | 745 | 0.62 | 0.009 | Down |
| Q569Z6 | Thrap3 | S | 253 | 0.44 | 0.000 | Down |
| Q569Z6 | Thrap3 | S | 264 | 0.49 | 0.010 | Down |
| Q569Z6 | Thrap3 | S | 248 | 0.64 | 0.007 | Down |
| Q569Z6 | Thrap3 | S | 695 | 0.64 | 0.003 | Down |
| Q5DW34 | Ehmt1 | S | 81 | 0.52 | 0.002 | Down |
| Q5HZI1 | Mtus1 | S | 894 | 0.35 | 0.000 | Down |
| Q5NBX1 | Cobl | S | 332 | 0.09 | 0.003 | Down |
| Q5NBX1 | Cobl | S | 1128 | 0.17 | 0.000 | Down |
| Q5NBX1 | Cobl | T | 824 | 0.43 | 0.040 | Down |
| Q5NBX1 | Cobl | S | 676 | 0.45 | 0.027 | Down |
| Q5PR69 | Kiaa1211 | S | 1016 | 0.66 | 0.016 | Down |
| Q5PR69 | Kiaa1211 | S | 487 | 0.66 | 0.016 | Down |
| Q5PR69 | Kiaa1211 | S | 992 | 0.66 | 0.003 | Down |
| Q5SWY7 | Fam83g | S | 649 | 0.35 | 0.012 | Down |
| Q5SYD0 | Myo1d | S | 486 | 0.26 | 0.001 | Down |
| Q5XJE5 | Leo1 | T | 189 | 0.58 | 0.000 | Down |
| Q5XJE5 | Leo1 | S | 262 | 0.61 | 0.015 | Down |
| Q60575 | Kif1b | S | 1075 | 0.64 | 0.012 | Down |
| Q60953 | Pml | T | 527 | 0.51 | 0.000 | Down |
| Q60953 | Pml | S | 515 | 0.54 | 0.002 | Down |
| Q60953 | Pml | S | 528 | 0.54 | 0.003 | Down |
| Q60974 | Ncor1 | S | 2329 | 0.54 | 0.000 | Down |
| Q60974 | Ncor1 | S | 2134 | 0.66 | 0.015 | Down |
| Q61069 | Usf1 | S | 186 | 0.66 | 0.002 | Down |
| Q61140 | Bcar1 | S | 643 | 0.50 | 0.002 | Down |
| Q61165 | Slc9a1 | S | 707 | 0.62 | 0.006 | Down |
| Q61233 | Lcp1 | S | 257 | 0.26 | 0.000 | Down |
| Q61391 | Mme | S | 4 | 0.27 | 0.000 | Down |
| Q61391 | Mme | S | 6 | 0.29 | 0.000 | Down |
| Q61469 | Ppap2a | S | 273 | 0.60 | 0.000 | Down |
| Q61703 | Itih2 | S | 60 | 0.52 | 0.000 | Down |
| Q61823 | Pdcd4 | T | 93 | 0.48 | 0.001 | Down |
| Q61823 | Pdcd4 | S | 94 | 0.52 | 0.017 | Down |
| Q62053 | Ptger2 | S | 138 | 0.26 | 0.005 | Down |
| Q62188 | Dpysl3 | T | 514 | 0.67 | 0.002 | Down |
| Q62219 | Tgfb1i1 | S | 194 | 0.43 | 0.003 | Down |
| Q62273 | Slc26a2 | S | 12 | 0.34 | 0.002 | Down |
| Q62417 | Sorbs1 | S | 58 | 0.63 | 0.017 | Down |
| Q62504 | Spen | S | 1293 | 0.62 | 0.019 | Down |
| Q62523 | Zyx | S | 336 | 0.52 | 0.001 | Down |
| Q63918 | Sdpr | S | 218 | 0.52 | 0.000 | Down |
| Q63ZV0 | Insm1 | T | 174 | 0.42 | 0.005 | Down |
| Q63ZV0 | Insm1 | S | 177 | 0.42 | 0.005 | Down |
| Q64442 | Sord | S | 169 | 0.38 | 0.002 | Down |
| Q64464 | Cyp3a13 | S | 501 | 0.52 | 0.001 | Down |
| Q6DIB4 | Nol4l | S | 458 | 0.48 | 0.012 | Down |
| Q6NXY9 | Polr3g | S | 158 | 0.51 | 0.004 | Down |
| Q6NZJ6 | Eif4g1 | S | 1211 | 0.60 | 0.002 | Down |
| Q6NZQ6 | Znf740 | S | 19 | 0.48 | 0.048 | Down |
| Q6P5B0 | Rrp12 | S | 894 | 0.48 | 0.002 | Down |
| Q6P6L0 | Filip1l | S | 789 | 0.47 | 0.001 | Down |
| Q6P6L0 | Filip1l | T | 992 | 0.47 | 0.003 | Down |
| Q6P9K8 | Caskin1 | S | 1363 | 0.62 | 0.002 | Down |
| Q6PAM1 | Txlna | S | 522 | 0.62 | 0.035 | Down |
| Q6PDN3 | Mylk | S | 1801 | 0.36 | 0.000 | Down |
| Q6PDN3 | Mylk | T | 1800 | 0.40 | 0.003 | Down |
| Q6PDN3 | Mylk | S | 1798 | 0.53 | 0.011 | Down |
| Q6PHZ2 | Camk2d | T | 337 | 0.59 | 0.001 | Down |
| Q6PIJ4 | Nfrkb | S | 176 | 0.38 | 0.022 | Down |
| Q6PIJ4 | Nfrkb | S | 180 | 0.38 | 0.022 | Down |
| Q6Y7W8 | Gigyf2 | S | 161 | 0.51 | 0.002 | Down |
| Q6ZQ58 | Larp1 | T | 503 | 0.35 | 0.005 | Down |
| Q6ZQB6 | Ppip5k2 | S | 499 | 0.26 | 0.003 | Down |
| Q70FJ1 | Akap9 | S | 3694 | 0.52 | 0.004 | Down |
| Q70IV5 | Synm | S | 172 | 0.18 | 0.000 | Down |
| Q70IV5 | Synm | S | 780 | 0.22 | 0.004 | Down |
| Q70IV5 | Synm | S | 1044 | 0.26 | 0.000 | Down |
| Q70IV5 | Synm | S | 1049 | 0.46 | 0.000 | Down |
| Q70IV5 | Synm | S | 1087 | 0.56 | 0.004 | Down |
| Q7TN37 | Trpm4 | S | 527 | 0.35 | 0.005 | Down |
| Q7TPV4 | Mybbp1a | S | 1164 | 0.60 | 0.001 | Down |
| Q80TJ1 | Cadps | S | 89 | 0.67 | 0.047 | Down |
| Q80TS3 | Lphn3 | S | 1508 | 0.65 | 0.021 | Down |
| Q80TS3 | Lphn3 | S | 1509 | 0.65 | 0.021 | Down |
| Q80U72 | Scrib | S | 821 | 0.60 | 0.016 | Down |
| Q80VC9 | Camsap3 | S | 767 | 0.61 | 0.002 | Down |
| Q80VP1 | Epn1 | S | 418 | 0.59 | 0.005 | Down |
| Q80WJ7 | Mtdh | T | 96 | 0.63 | 0.002 | Down |
| Q80XC3 | Usp6nl | T | 570 | 0.49 | 0.017 | Down |
| Q80XC3 | Usp6nl | S | 574 | 0.56 | 0.014 | Down |
| Q80Y61 | Baiap2l2 | S | 227 | 0.05 | 0.002 | Down |
| Q80Y61 | Baiap2l2 | S | 225 | 0.06 | 0.002 | Down |
| Q80Y61 | Baiap2l2 | S | 272 | 0.27 | 0.000 | Down |
| Q80Y61 | Baiap2l2 | S | 459 | 0.35 | 0.003 | Down |
| Q80YN3 | Bcas1 | S | 443 | 0.66 | 0.005 | Down |
| Q812A2 | Srgap3 | S | 858 | 0.61 | 0.049 | Down |
| Q8BGB2 | Ttc7a | S | 648 | 0.44 | 0.013 | Down |
| Q8BGD9 | Eif4b | S | 422 | 0.17 | 0.004 | Down |
| Q8BH64 | Ehd2 | S | 438 | 0.62 | 0.013 | Down |
| Q8BHD7 | Ptbp3 | S | 27 | 0.31 | 0.009 | Down |
| Q8BHJ9 | Slu7 | S | 215 | 0.64 | 0.001 | Down |
| Q8BIE6 | Frmd4a | T | 964 | 0.09 | 0.007 | Down |
| Q8BIE6 | Frmd4a | S | 965 | 0.09 | 0.007 | Down |
| Q8BP47 | Nars | S | 493 | 0.65 | 0.050 | Down |
| Q8BTI8 | Srrm2 | S | 2660 | 0.39 | 0.000 | Down |
| Q8BTI8 | Srrm2 | T | 2362 | 0.43 | 0.002 | Down |
| Q8BTI8 | Srrm2 | S | 2656 | 0.51 | 0.000 | Down |
| Q8BTI8 | Srrm2 | S | 2648 | 0.54 | 0.009 | Down |
| Q8BTI8 | Srrm2 | S | 2351 | 0.54 | 0.000 | Down |
| Q8BTM8 | Flna | S | 2152 | 0.38 | 0.000 | Down |
| Q8BTW9 | Pak4 | S | 104 | 0.58 | 0.015 | Down |
| Q8BU14 | Sec62 | S | 341 | 0.65 | 0.008 | Down |
| Q8BX57 | Pxk | S | 452 | 0.49 | 0.029 | Down |
| Q8BZF8 | Pgm5 | S | 122 | 0.29 | 0.000 | Down |
| Q8BZR9 | unknown | S | 25 | 0.64 | 0.027 | Down |
| Q8C0N2 | Agpat9 | S | 68 | 0.41 | 0.000 | Down |
| Q8C1D8 | Iws1 | S | 248 | 0.58 | 0.004 | Down |
| Q8C1D8 | Iws1 | S | 261 | 0.58 | 0.004 | Down |
| Q8C1D8 | Iws1 | S | 384 | 0.61 | 0.009 | Down |
| Q8C1D8 | Iws1 | S | 386 | 0.61 | 0.009 | Down |
| Q8C3W1 | unknown | S | 278 | 0.63 | 0.001 | Down |
| Q8CC35 | Synpo | S | 227 | 0.48 | 0.005 | Down |
| Q8CC35 | Synpo | S | 230 | 0.48 | 0.005 | Down |
| Q8CGA3 | Slc43a2 | S | 552 | 0.53 | 0.006 | Down |
| Q8CHC4 | Synj1 | S | 1147 | 0.65 | 0.029 | Down |
| Q8CI51 | Pdlim5 | S | 228 | 0.36 | 0.001 | Down |
| Q8CI59 | Steap3 | S | 17 | 0.56 | 0.000 | Down |
| Q8CIW6 | Slc26a6 | S | 751 | 0.41 | 0.005 | Down |
| Q8JZQ9 | Eif3b | Y | 328 | 0.17 | 0.002 | Down |
| Q8K0T0 | Rtn1 | S | 350 | 0.54 | 0.045 | Down |
| Q8K4L4 | Pof1b | S | 151 | 0.38 | 0.029 | Down |
| Q8K4P0 | Wdr33 | S | 1204 | 0.66 | 0.010 | Down |
| Q8R000 | Slc51a | S | 330 | 0.04 | 0.005 | Down |
| Q8R0S2 | Iqsec1 | S | 251 | 0.62 | 0.028 | Down |
| Q8R0W0 | Eppk1 | S | 2606 | 0.60 | 0.000 | Down |
| Q8R149 | Bud13 | T | 144 | 0.54 | 0.002 | Down |
| Q8R5F7 | Ifih1 | S | 302 | 0.30 | 0.000 | Down |
| Q8R5H1 | Usp15 | S | 229 | 0.46 | 0.006 | Down |
| Q8VCE2 | Gpn1 | S | 314 | 0.60 | 0.001 | Down |
| Q8VDD5 | Myh9 | S | 1943 | 0.59 | 0.006 | Down |
| Q8VDF3 | Dapk2 | S | 349 | 0.29 | 0.006 | Down |
| Q8VDF3 | Dapk2 | S | 299 | 0.58 | 0.001 | Down |
| Q8VDJ3 | Hdlbp | S | 31 | 0.63 | 0.015 | Down |
| Q8VDM1 | Zgpat | Y | 168 | 0.55 | 0.027 | Down |
| Q8VDM1 | Zgpat | Y | 170 | 0.55 | 0.027 | Down |
| Q8VDM1 | Zgpat | T | 172 | 0.55 | 0.027 | Down |
| Q8VDP3 | Mical1 | S | 777 | 0.47 | 0.000 | Down |
| Q8VE65 | Taf12 | S | 51 | 0.62 | 0.002 | Down |
| Q8VE88 | Fam114a2 | S | 119 | 0.66 | 0.004 | Down |
| Q8VEL9 | Rem2 | S | 296 | 0.52 | 0.036 | Down |
| Q8VHF2 | Cdhr5 | S | 755 | 0.27 | 0.000 | Down |
| Q8VHF2 | Cdhr5 | S | 753 | 0.45 | 0.001 | Down |
| Q8VHW2 | Cacng8 | S | 290 | 0.65 | 0.026 | Down |
| Q91WK0 | Lrrfip2 | S | 111 | 0.66 | 0.000 | Down |
| Q91WL0 | Eps8l3 | S | 428 | 0.10 | 0.002 | Down |
| Q91WL0 | Eps8l3 | S | 427 | 0.10 | 0.000 | Down |
| Q91WL0 | Eps8l3 | S | 238 | 0.43 | 0.001 | Down |
| Q91WL0 | Eps8l3 | S | 417 | 0.60 | 0.003 | Down |
| Q91WL0 | Eps8l3 | S | 214 | 0.62 | 0.001 | Down |
| Q91WV7 | Slc3a1 | S | 14 | 0.30 | 0.001 | Down |
| Q91XA2 | Golm1 | S | 213 | 0.51 | 0.011 | Down |
| Q91XC8 | Dap | T | 50 | 0.63 | 0.012 | Down |
| Q91XU0 | Wrnip1 | S | 75 | 0.40 | 0.001 | Down |
| Q91YE8 | Synpo2 | S | 300 | 0.12 | 0.000 | Down |
| Q91YE8 | Synpo2 | S | 895 | 0.37 | 0.001 | Down |
| Q91Z58 | unknown | S | 368 | 0.30 | 0.001 | Down |
| Q922D4 | Ppp6r3 | T | 602 | 0.63 | 0.031 | Down |
| Q99J27 | Slc33a1 | S | 42 | 0.53 | 0.001 | Down |
| Q99JG3 | Anxa13 | S | 10 | 0.47 | 0.004 | Down |
| Q99K01 | Pdxdc1 | T | 687 | 0.45 | 0.000 | Down |
| Q99K30 | Eps8l2 | S | 242 | 0.04 | 0.000 | Down |
| Q99K30 | Eps8l2 | S | 573 | 0.36 | 0.000 | Down |
| Q99K30 | Eps8l2 | T | 575 | 0.44 | 0.002 | Down |
| Q99LX5 | Mmtag2 | S | 232 | 0.67 | 0.018 | Down |
| Q99MK8 | Adrbk1 | S | 670 | 0.63 | 0.010 | Down |
| Q99MZ6 | Myo7b | S | 622 | 0.16 | 0.000 | Down |
| Q9CQ36 | Pole4 | T | 11 | 0.51 | 0.002 | Down |
| Q9CQS8 | Sec61b | S | 17 | 0.67 | 0.047 | Down |
| Q9CR86 | Carhsp1 | S | 42 | 0.40 | 0.012 | Down |
| Q9CR86 | Carhsp1 | S | 31 | 0.47 | 0.003 | Down |
| Q9CR86 | Carhsp1 | S | 53 | 0.63 | 0.002 | Down |
| Q9CX86 | Hnrnpa0 | S | 84 | 0.56 | 0.002 | Down |
| Q9CY58 | Serbp1 | S | 234 | 0.57 | 0.003 | Down |
| Q9CYS6 | unknown | S | 14 | 0.59 | 0.040 | Down |
| Q9D279 | Misp | T | 194 | 0.27 | 0.000 | Down |
| Q9D279 | Misp | S | 364 | 0.40 | 0.002 | Down |
| Q9D279 | Misp | S | 346 | 0.43 | 0.001 | Down |
| Q9D279 | Misp | S | 77 | 0.43 | 0.045 | Down |
| Q9D281 | Fam114a1 | T | 197 | 0.46 | 0.004 | Down |
| Q9D2D7 | Znf687 | S | 228 | 0.54 | 0.009 | Down |
| Q9D5V6 | Syap1 | T | 262 | 0.64 | 0.001 | Down |
| Q9D620 | Rab11fip1 | S | 383 | 0.57 | 0.015 | Down |
| Q9D8S3 | Arfgap3 | Y | 363 | 0.25 | 0.009 | Down |
| Q9D8T7 | Slirp | T | 104 | 0.62 | 0.019 | Down |
| Q9D8T7 | Slirp | S | 105 | 0.64 | 0.022 | Down |
| Q9DBJ3 | Baiap2l1 | S | 421 | 0.44 | 0.004 | Down |
| Q9DBR7 | Ppp1r12a | S | 870 | 0.40 | 0.001 | Down |
| Q9EQU5 | Set | S | 30 | 0.33 | 0.000 | Down |
| Q9ERG0 | Lima1 | S | 607 | 0.52 | 0.005 | Down |
| Q9ERG0 | Lima1 | S | 488 | 0.58 | 0.001 | Down |
| Q9ES64 | Ush1c | S | 201 | 0.13 | 0.001 | Down |
| Q9ESN9 | Mapk8ip3 | S | 564 | 0.44 | 0.001 | Down |
| Q9EST5 | Anp32b | T | 265 | 0.21 | 0.012 | Down |
| Q9ESU6 | Brd4 | S | 1153 | 0.35 | 0.005 | Down |
| Q9ET54 | Palld | S | 1129 | 0.19 | 0.009 | Down |
| Q9ET54 | Palld | S | 901 | 0.39 | 0.001 | Down |
| Q9ET77 | Jph3 | S | 228 | 0.19 | 0.008 | Down |
| Q9ET77 | Jph3 | S | 232 | 0.19 | 0.008 | Down |
| Q9ET77 | Jph3 | S | 233 | 0.19 | 0.008 | Down |
| Q9ET77 | Jph3 | S | 236 | 0.19 | 0.008 | Down |
| Q9ET77 | Jph3 | S | 239 | 0.19 | 0.008 | Down |
| Q9ET78 | Jph2 | S | 462 | 0.43 | 0.010 | Down |
| Q9JHU2 | Palmd | S | 384 | 0.60 | 0.022 | Down |
| Q9JIK5 | Ddx21 | S | 118 | 0.49 | 0.002 | Down |
| Q9JIX8 | Acin1 | S | 479 | 0.31 | 0.004 | Down |
| Q9JIX8 | Acin1 | S | 583 | 0.45 | 0.032 | Down |
| Q9JIX8 | Acin1 | S | 585 | 0.45 | 0.032 | Down |
| Q9JIX8 | Acin1 | S | 491 | 0.56 | 0.004 | Down |
| Q9JIX8 | Acin1 | T | 487 | 0.66 | 0.010 | Down |
| Q9JJ89 | Ccdc86 | S | 161 | 0.60 | 0.001 | Down |
| Q9JKA5 | Gpa33 | S | 312 | 0.27 | 0.005 | Down |
| Q9JKA5 | Gpa33 | T | 313 | 0.27 | 0.005 | Down |
| Q9JKA5 | Gpa33 | S | 308 | 0.28 | 0.001 | Down |
| Q9QXA6 | Slc7a9 | S | 18 | 0.20 | 0.000 | Down |
| Q9QXY1 | Tjp3 | S | 195 | 0.29 | 0.000 | Down |
| Q9QXY1 | Tjp3 | S | 111 | 0.56 | 0.006 | Down |
| Q9QXY1 | Tjp3 | S | 343 | 0.61 | 0.001 | Down |
| Q9QY06 | Myo9b | S | 1264 | 0.65 | 0.005 | Down |
| Q9QY23 | Pkp3 | S | 314 | 0.29 | 0.007 | Down |
| Q9QYX7 | Pclo | S | 4286 | 0.61 | 0.030 | Down |
| Q9QZK7 | Dok3 | S | 274 | 0.63 | 0.009 | Down |
| Q9WTV7 | Rlim | S | 78 | 0.65 | 0.003 | Down |
| Q9WUH1 | Tmem115 | T | 318 | 0.66 | 0.021 | Down |
| Q9WV92 | Epb41l3 | S | 804 | 0.59 | 0.003 | Down |
| Q9WVA4 | Tagln2 | S | 163 | 0.56 | 0.030 | Down |
| Q9Z0G9 | Cldn3 | S | 202 | 0.51 | 0.003 | Down |
| Q9Z0S5 | Cldn15 | T | 210 | 0.18 | 0.002 | Down |
| Q9Z0S5 | Cldn15 | S | 211 | 0.40 | 0.001 | Down |
| Q9Z0S5 | Cldn15 | S | 217 | 0.40 | 0.001 | Down |
| Q9Z0U1 | Tjp2 | S | 420 | 0.22 | 0.000 | Down |
| Q9Z0U1 | Tjp2 | S | 947 | 0.42 | 0.001 | Down |
| Q9Z0U1 | Tjp2 | S | 948 | 0.42 | 0.001 | Down |
| Q9Z0U1 | Tjp2 | S | 421 | 0.45 | 0.000 | Down |
| Q9Z0U1 | Tjp2 | S | 968 | 0.55 | 0.004 | Down |
| Q9Z0Z4 | Heph | S | 1144 | 0.20 | 0.011 | Down |
| Q9Z0Z4 | Heph | S | 1149 | 0.36 | 0.001 | Down |
| Q9Z1B7 | Mapk13 | Y | 182 | 0.24 | 0.000 | Down |
| Q9Z277 | Baz1b | S | 1338 | 0.66 | 0.026 | Down |
| Q9Z2N8 | Actl6a | S | 233 | 0.60 | 0.022 | Down |
| A2A699 | Fam171a2 | S | 739 | 1.55 | 0.002 | Up |
| B2RUJ5 | Apba1 | S | 391 | 2.02 | 0.013 | Up |
| D3YVF0 | Akap5 | S | 22 | 2.18 | 0.002 | Up |
| D3YZU1 | Shank1 | T | 2 | 1.55 | 0.018 | Up |
| P04370 | Mbp | T | 226 | 1.63 | 0.009 | Up |
| P04370 | Mbp | T | 229 | 1.68 | 0.002 | Up |
| P06837 | Gap43 | S | 144 | 1.73 | 0.019 | Up |
| P11031 | Sub1 | S | 17 | 1.58 | 0.021 | Up |
| P11798 | Camk2a | S | 272 | 3.56 | 0.000 | Up |
| P14873 | Map1b | S | 1616 | 1.75 | 0.001 | Up |
| P16054 | Prkce | S | 729 | 2.02 | 0.000 | Up |
| P19246 | Nefh | S | 703 | 1.50 | 0.000 | Up |
| P19246 | Nefh | S | 727 | 1.50 | 0.009 | Up |
| P19246 | Nefh | S | 739 | 1.51 | 0.004 | Up |
| P19246 | Nefh | S | 679 | 1.52 | 0.014 | Up |
| P19246 | Nefh | S | 583 | 1.73 | 0.008 | Up |
| P19246 | Nefh | S | 571 | 1.77 | 0.001 | Up |
| P20357 | Map2 | T | 1358 | 1.96 | 0.006 | Up |
| P28652 | Camk2b | S | 280 | 1.69 | 0.001 | Up |
| P35564 | Canx | S | 563 | 8.49 | 0.005 | Up |
| P35802 | Gpm6a | S | 270 | 1.53 | 0.036 | Up |
| P42859 | Htt | S | 1841 | 1.68 | 0.015 | Up |
| P47746 | Cnr1 | S | 442 | 2.08 | 0.000 | Up |
| P47746 | Cnr1 | T | 440 | 2.09 | 0.002 | Up |
| P51830 | Adcy9 | S | 354 | 1.59 | 0.008 | Up |
| P51830 | Adcy9 | S | 357 | 1.59 | 0.008 | Up |
| P56695 | Wfs1 | S | 71 | 3.12 | 0.000 | Up |
| P59281 | Arhgap39 | S | 115 | 1.79 | 0.002 | Up |
| P60202 | Plp1 | S | 134 | 1.59 | 0.015 | Up |
| P62774 | Mtpn | T | 31 | 1.56 | 0.005 | Up |
| P62996 | Tra2b | T | 201 | 1.56 | 0.033 | Up |
| P63141 | Kcna2 | S | 441 | 1.75 | 0.001 | Up |
| P63318 | Prkcg | T | 514 | 1.83 | 0.015 | Up |
| P63328 | Ppp3ca | S | 469 | 1.52 | 0.001 | Up |
| P63328 | Ppp3ca | T | 468 | 1.65 | 0.007 | Up |
| P67871 | Csnk2b | S | 209 | 1.70 | 0.017 | Up |
| P68181 | Prkacb | T | 198 | 1.87 | 0.001 | Up |
| P68373 | Tuba1c | S | 48 | 1.75 | 0.003 | Up |
| P68373 | Tuba1c | T | 51 | 1.90 | 0.001 | Up |
| P70349 | Hint1 | S | 45 | 1.56 | 0.013 | Up |
| P97792 | Cxadr | S | 332 | 1.98 | 0.000 | Up |
| Q00PI9 | Hnrnpul2 | S | 183 | 1.65 | 0.012 | Up |
| Q03517 | Scg2 | S | 176 | 1.55 | 0.001 | Up |
| Q04447 | Ckb | T | 35 | 1.73 | 0.000 | Up |
| Q3UNH4 | Gprin1 | T | 493 | 1.59 | 0.009 | Up |
| Q3UNH4 | Gprin1 | S | 771 | 3.58 | 0.016 | Up |
| Q4JIM5 | Abl2 | S | 936 | 2.16 | 0.000 | Up |
| Q52KI8 | Srrm1 | S | 714 | 1.82 | 0.003 | Up |
| Q52KI8 | Srrm1 | S | 713 | 5.96 | 0.000 | Up |
| Q5DTJ9 | Mypn | S | 641 | 1.63 | 0.034 | Up |
| Q5DTL9 | Slc4a10 | S | 1116 | 2.34 | 0.002 | Up |
| Q5GH67 | Xkr4 | S | 522 | 1.61 | 0.024 | Up |
| Q5U4C3 | Scaf1 | S | 922 | 1.54 | 0.000 | Up |
| Q5U4C3 | Scaf1 | T | 923 | 1.54 | 0.000 | Up |
| Q5U4C3 | Scaf1 | S | 682 | 3.32 | 0.000 | Up |
| Q61206 | Pafah1b2 | S | 64 | 1.56 | 0.011 | Up |
| Q61234 | Snta1 | S | 194 | 1.99 | 0.000 | Up |
| Q61234 | Snta1 | S | 195 | 2.25 | 0.005 | Up |
| Q6IR34 | Gpsm1 | S | 491 | 2.05 | 0.001 | Up |
| Q6P542 | Abcf1 | S | 194 | 3.14 | 0.003 | Up |
| Q6P5G0 | Mapk4 | S | 504 | 1.52 | 0.015 | Up |
| Q6PER3 | Mapre3 | S | 162 | 1.69 | 0.012 | Up |
| Q6VNS1 | Ntrk3 | S | 561 | 1.55 | 0.001 | Up |
| Q6ZPF3 | Tiam2 | T | 1606 | 5.71 | 0.000 | Up |
| Q6ZQ18 | Efr3b | S | 709 | 1.65 | 0.005 | Up |
| Q7TQA1 | Igsf1 | S | 1275 | 1.82 | 0.003 | Up |
| Q80XU3 | Nucks1 | S | 214 | 1.51 | 0.006 | Up |
| Q8BG40 | Katnb1 | S | 363 | 2.03 | 0.002 | Up |
| Q8BGH4 | Reep1 | S | 152 | 1.57 | 0.017 | Up |
| Q8BWS5 | Gprin3 | S | 323 | 1.57 | 0.010 | Up |
| Q8BWS5 | Gprin3 | S | 325 | 1.62 | 0.017 | Up |
| Q8BX17 | Gemin5 | S | 1414 | 1.67 | 0.015 | Up |
| Q8CC35 | Synpo | S | 258 | 1.51 | 0.000 | Up |
| Q8CCJ4 | Amer2 | T | 282 | 1.75 | 0.001 | Up |
| Q8CCJ4 | Amer2 | S | 244 | 3.15 | 0.001 | Up |
| Q8CG79 | Tp53bp2 | S | 697 | 1.79 | 0.007 | Up |
| Q8CJ19 | Mical3 | S | 1187 | 1.85 | 0.004 | Up |
| Q8K212 | Pacs1 | S | 526 | 1.51 | 0.033 | Up |
| Q8R3Z5 | Cacnb1 | T | 205 | 1.56 | 0.003 | Up |
| Q91V14 | Slc12a5 | T | 1029 | 1.62 | 0.009 | Up |
| Q91W82 | Ube2e2 | S | 18 | 1.58 | 0.002 | Up |
| Q99JP6 | Homer3 | S | 159 | 1.86 | 0.034 | Up |
| Q99MS8 | Tpgs1 | S | 279 | 2.88 | 0.012 | Up |
| Q99MX7 | Cecr6 | S | 70 | 1.57 | 0.046 | Up |
| Q99NF2 | Nsmf | S | 138 | 2.53 | 0.010 | Up |
| Q9CR57 | Rpl14 | S | 139 | 2.59 | 0.012 | Up |
| Q9D415 | Dlgap1 | S | 509 | 1.59 | 0.001 | Up |
| Q9JI08 | Bin3 | S | 48 | 1.64 | 0.007 | Up |
| Q9JI08 | Bin3 | T | 49 | 1.64 | 0.007 | Up |
| Q9JI08 | Bin3 | S | 56 | 1.64 | 0.007 | Up |
| Q9JJV4 | Cacng4 | S | 253 | 1.93 | 0.027 | Up |
| Q9JMG7 | Hdgfrp3 | S | 179 | 1.55 | 0.012 | Up |
| Q9QWI6 | Srcin1 | S | 1127 | 1.55 | 0.002 | Up |
| Q9QXY6 | Ehd3 | S | 456 | 1.55 | 0.017 | Up |
| Q9QYB8 | Add2 | S | 620 | 2.36 | 0.003 | Up |
| Q9QYR6 | Map1a | S | 1205 | 1.61 | 0.009 | Up |
| Q9QYR6 | Map1a | S | 1208 | 1.67 | 0.001 | Up |
| Q9R1V6 | Adam22 | S | 864 | 1.96 | 0.025 | Up |
| Q9WV92 | Epb41l3 | S | 531 | 1.60 | 0.002 | Up |
| Q9Z2H5 | Epb41l1 | S | 671 | 1.69 | 0.025 | Up |
| Q9Z2H5 | Epb41l1 | S | 648 | 2.11 | 0.002 | Up |
| Q9Z2H5 | Epb41l1 | S | 650 | 2.78 | 0.000 | Up |
| Q9Z2Q6 | Sept5 | S | 225 | 1.53 | 0.010 | Up |

**Table S3.** Top ranked identified consensus motifs on significant phosphopeptides in the hippocampus of GF and FMT models with kinases predicted to target these sequences

| **Motifs** | **Kinase** | **Motif Score** | **Matches** | **Fold Increase** | |
| --- | --- | --- | --- | --- | --- |
| **Germ-free mice model** | | | | |  |
| **...R..s......** | CaMK II, PKA, PKC | 16.00 | 105 | 6.2 | |
| **......sP.....** | GSK-3, ERK1/2, CDK5 | 16.00 | 80 | 6.0 | |
| **......s.E....** | CK2 | 8.65 | 26 | 3.8 | |
| **......s.D....** | CK2 | 8.52 | 20 | 4.8 | |
| **Gut microbiota remodeling mice model** | | | | |  |
| **......sP.....** | GSK-3, ERK1/2, CDK5 | 16.00 | 187 | 6.3 | |
| **...R..s......** | CaMK II, PKA, PKC | 16.00 | 66 | 5.1 | |
| **......s..E...** | CK2 | 13.76 | 43 | 3.8 | |
| **......tP.....** | GSK-3, ERK1/2, CDK5 | 14.02 | 26 | 6.2 | |

**Table S4.** The common phosphoproteins between GF and FMT models

| **Protein accession** | **Gene symbol** | **Protein description** | **Phosphorylation residues and regulated type** | |
| --- | --- | --- | --- | --- |
|  |  |  | **GF mice model** | **FMT mice model** |
| D3YVF0 | Akap5 | A-kinase anchor protein 5 | S68↓ | S22↑ |
| O35691 | Pnn | Pinin | S100↑ | S68↓ |
| **P04370** | Mbp | Myelin basic protein | **T229↓**, S245↓ | T226↑, **T229↑** |
| **P06837** | Gap43 | Neuromodulin | T95↓, S128↓, S142↓, **S144↓** | **S144↑** |
| P11798 | Camk2a | Calcium/calmodulin-dependent protein kinase type II subunit alpha | T276↓ | S272↑ |
| P14873 | Map1b | Microtubule-associated protein 1B | S989↑, S1627↓, T1629↓, S2068↓ | S1616↑ |
| P16054 | Prkce | Protein kinase C epsilon type | T710↑ | S729↑ |
| P20357 | Map2 | Microtubule-associated protein 2 | S1013↓ | T1358↑, S1487↓ |
| P39447 | Tjp1 | Tight junction protein ZO-1 | S311↓, S313↓, S315↓ | S277↓, S280↓, S912↓ |
| **P47746** | Cnr1 | Cannabinoid receptor 1 | **T440↓, S442↓** | **T440↑, S442↑** |
| P51830 | Adcy9 | Adenylate cyclase type 9 | S365↓ | S354↑, S357↑ |
| P58871 | Tnks1bp1 | 182 kDa tankyrase-1-binding protein | S178↑ | S1657↓ |
| P59281 | Arhgap39 | Rho GTPase-activating protein 39 | S123↓ | S115↑ |
| P59648 | Fxyd7 | FXYD domain-containing ion transport regulator 7 | S56↓, S60↓ | S72↓ |
| P62996 | Tra2b | Transformer-2 protein homolog beta | S20↑, S22↑ | T30↓, T201↑ |
| P70255 | Nfic | Nuclear factor 1 C-type | S194↓ | S323↓ |
| P70336 | Rock2 | Rho-associated protein kinase 2 | S1052↓ | S549↓, S550↓, S553↓, T554↓ |
| Q00PI9 | Hnrnpul2 | Heterogeneous nuclear ribonucleoprotein U-like protein 2 | S159↑, S191↓ | S183↑ |
| **Q3TLH4** | Prrc2c | Protein PRRC2C | **S899↓** | **S899↓** |
| Q3UNH4 | Gprin1 | G protein-regulated inducer of neurite outgrowth 1 | S620↓, S622↓ | T493↑, S771↑ |
| Q3UYV9 | Ncbp1 | Nuclear cap-binding protein subunit 1 | S674↓ | S7↓ |
| Q4ACU6 | Shank3 | SH3 and multiple ankyrin repeat domains protein 3 | S781↓ | S482↓ |
| Q52KI8 | Srrm1 | Serine/arginine repetitive matrix protein 1 | T414↓ | S713↑, S714↑, S743↓, S745↓ |
| Q5GH67 | Xkr4 | XK-related protein 4 | S214↓ | S522↑ |
| **Q61234** | Snta1 | Alpha-1-syntrophin | **S194↓** | **S194↑**, S195↑ |
| **Q62188** | Dpysl3 | Dihydropyrimidinase-related protein 3 | **T514↓** | **T514↓** |
| Q6NZJ6 | Eif4g1 | Eukaryotic translation initiation factor 4 gamma 1 | S1597↓ | S1211↓ |
| **Q6PER3** | Mapre3 | Microtubule-associated protein RP/EB family member 3 | T161↓, **S162↓** | **S162↑** |
| **Q6ZQ18** | Efr3b | Protein EFR3 homolog B | **S709↓** | **S709↑** |
| Q6ZQ58 | Larp1 | La-related protein 1 | S494↓ | T503↓ |
| Q80XU3 | Nucks1 | Nuclear ubiquitous casein and cyclin-dependent kinase substrate 1 | S19↓ | S214↑ |
| Q80YN3 | Bcas1 | Breast carcinoma-amplified sequence 1 homolog | S601↓ | S443↓ |
| Q812A2 | Srgap3 | SLIT-ROBO Rho GTPase-activating protein 3 | S948↓, S954↑ | S858↓ |
| **Q8BGD9** | Eif4b | Eukaryotic translation initiation factor 4B | **S422↓** | **S422↓** |
| **Q8BTI8** | Srrm2 | Serine/arginine repetitive matrix protein 2 | T877↓, S882↓, Y1776↓, S1822↓, S1825↓, **S2656↓** | S2351↓, T2362↓, S2648↓, **S2656↓**, S2660↓ |
| **Q8BX57** | Pxk | PX domain-containing protein kinase-like protein | S448↓, **S452↓** | **S452↓** |
| **Q8K212** | Pacs1 | Phosphofurin acidic cluster sorting protein 1 | **S526↓** | **S526↑** |
| Q8R149 | Bud13 | BUD13 homolog | T262↓, S272↓ | T144↓ |
| Q8VDJ3 | Hdlbp | Vigilin | S944↓ | S31↓ |
| Q8VHW2 | Cacng8 | Voltage-dependent calcium channel gamma-8 subunit | S237↓, S277↓, S280↓ | S290↓ |
| Q91V14 | Slc12a5 | Solute carrier family 12 member 5 | S27↓ | T1029↑ |
| **Q9ESN9** | Mapk8ip3 | C-Jun-amino-terminal kinase-interacting protein 3 | **S564↓**, S755↓ | **S564↓** |
| **Q9EST5** | Anp32b | Acidic leucine-rich nuclear phosphoprotein 32 family member B | **T265↓** | **T265↓** |
| Q9QYB8 | Add2 | Beta-adducin | S614↓, S618↓ | S620↑ |
| Q9QYR6 | Map1a | Microtubule-associated protein 1A | S611↓, T1633↓, S1634↓ | S1205↑, S1208↑ |
| Q9QYX7 | Pclo | Protein piccolo | T1390↓ | S4286↓ |


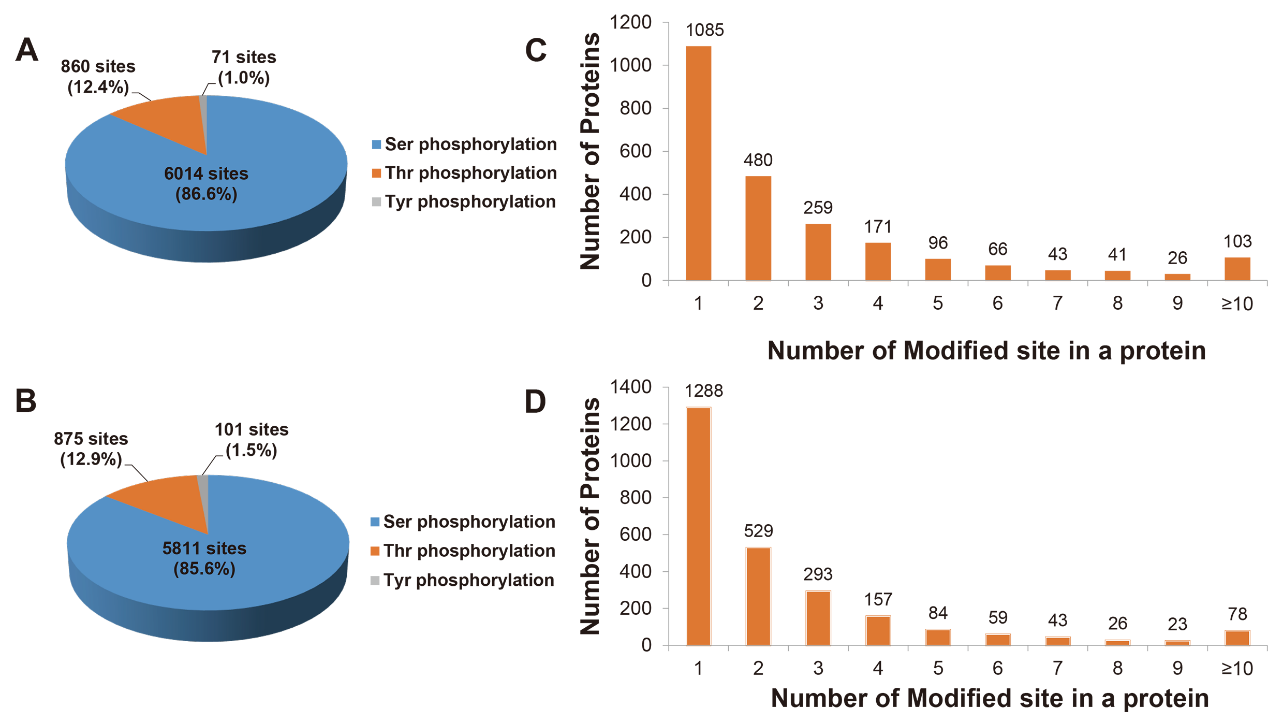


**Figure S1.** Global phosphoproteomic analysis of the hippocampus in GF and FMT models. (A and B) Pie chart depicting the phosphorylation sites quantified in GF and FMT mice models, respectively. (C and D) The number of modified sites within each phosphoprotein in GF and FMT mice models, respectively.


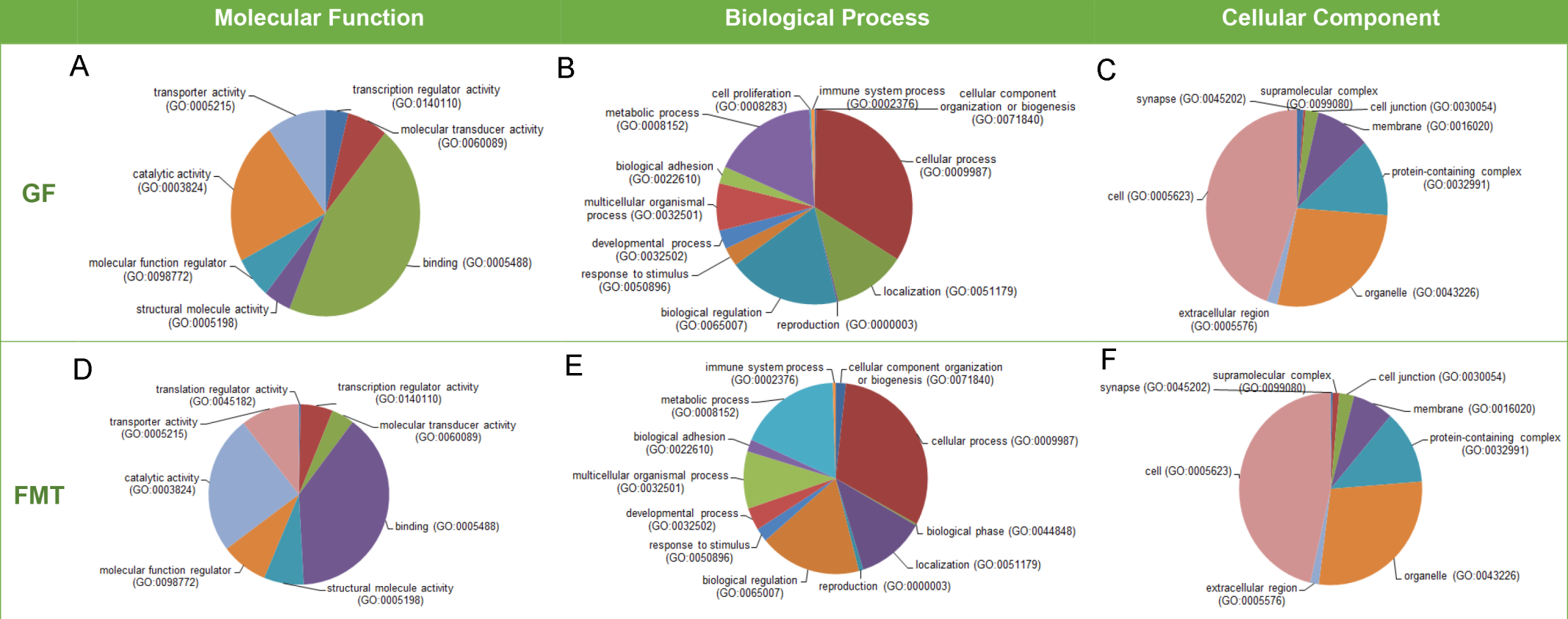


**Figure S2.** Gene Ontology (GO) annotations for the significant phosphoproteins in GF and FMT models.


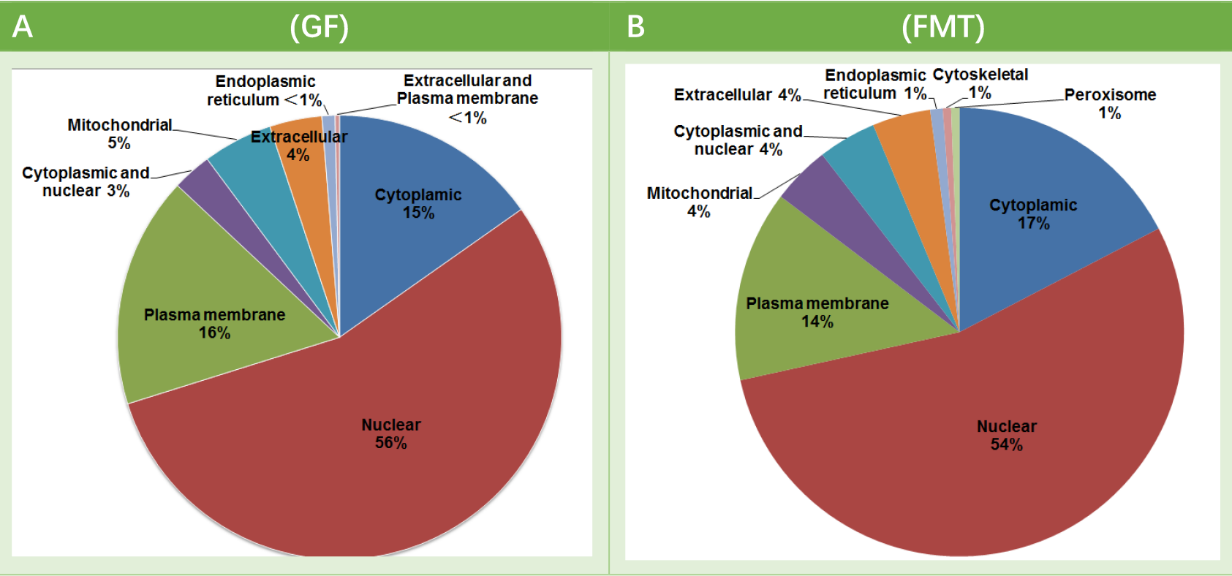


**Figure S3.** The subcellular location of significant phosphoproteins in GF and FMT models.


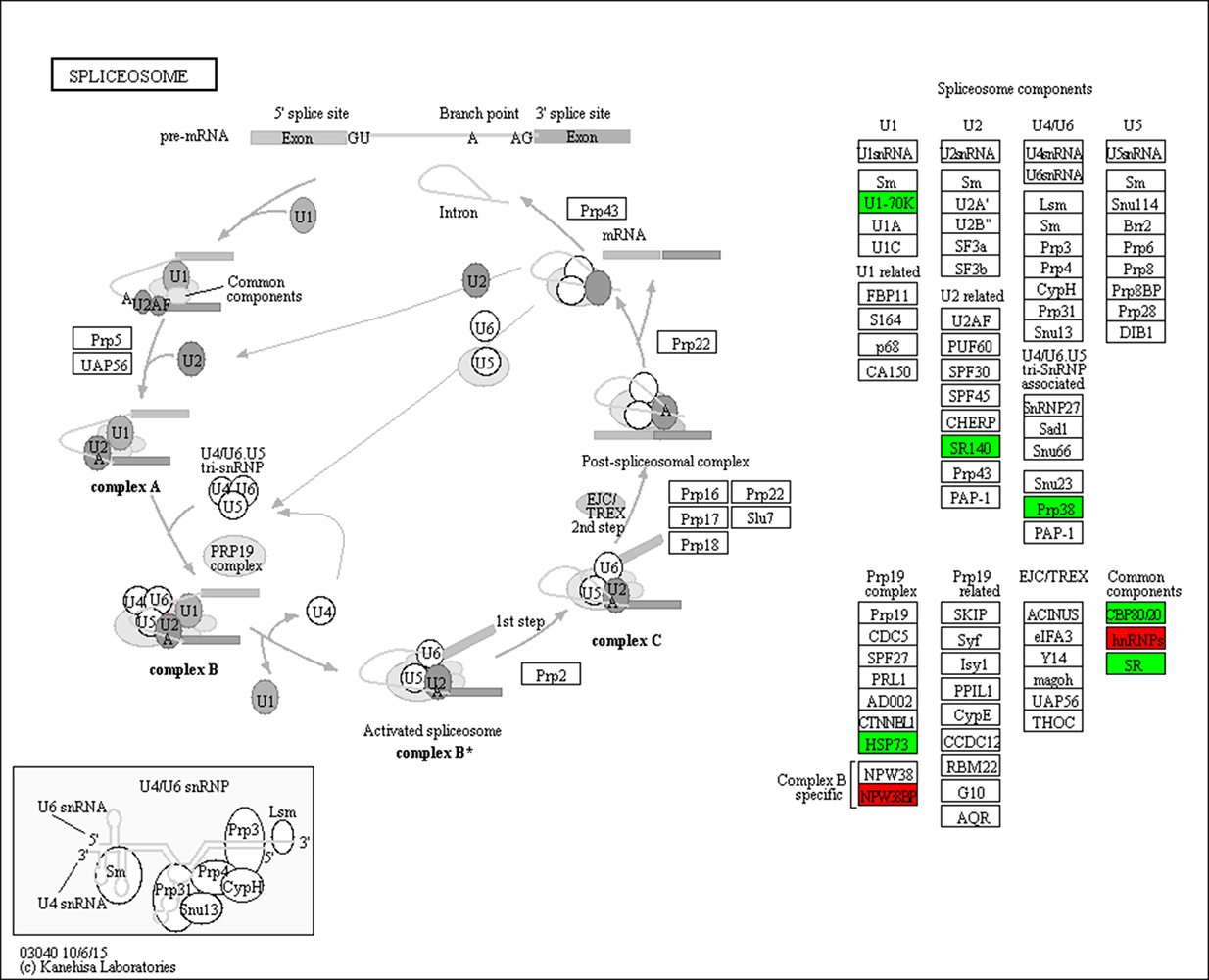


**Figure S4.** Construction of the spliceosome pathway enriched from KEGG pathway analysis.

Proteins in red were up-regulated phosphorylation in specific residues while those in green were down-regulated in germ-free (GF) VS specific-pathogen-free (SPF) mice.


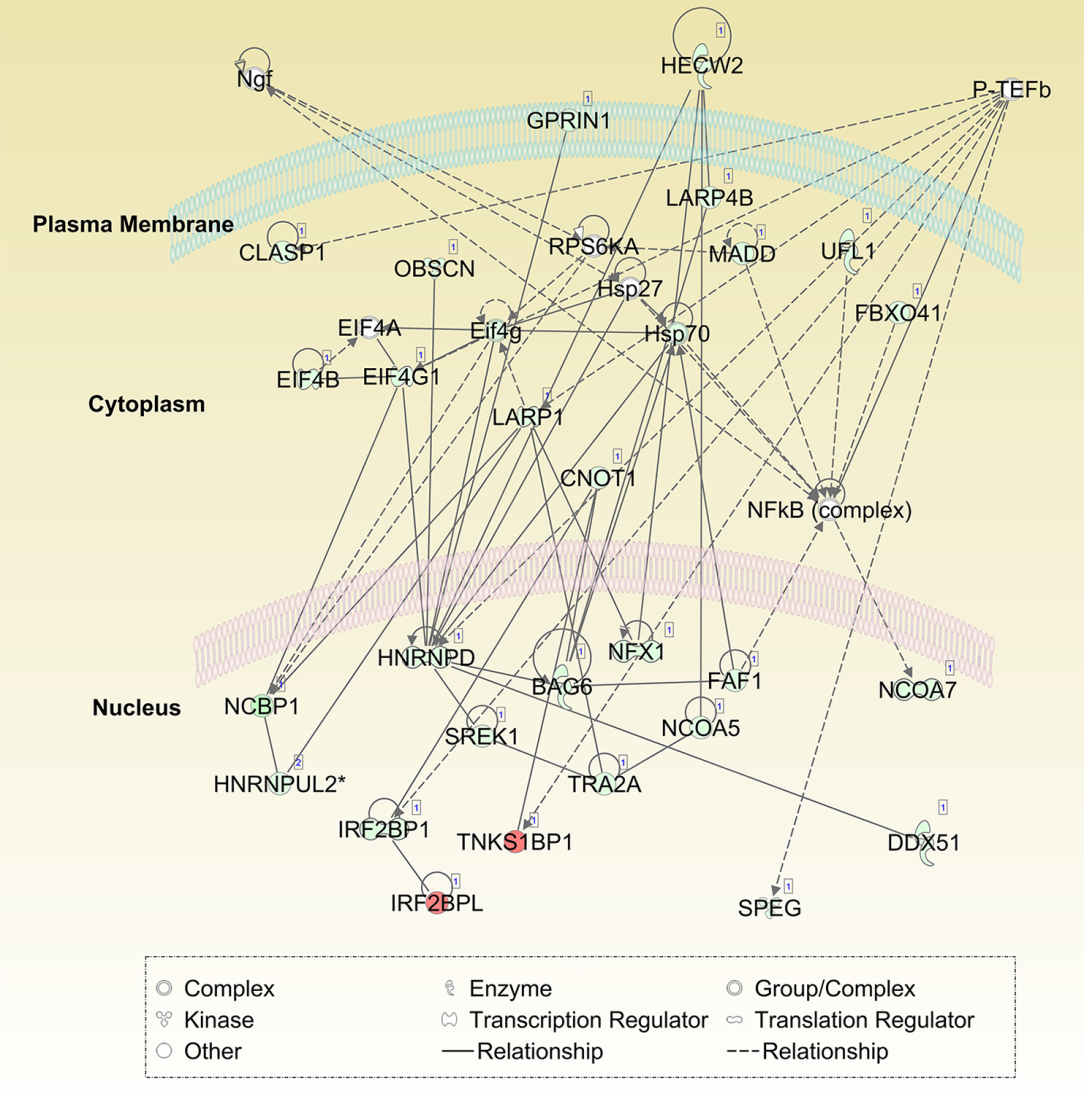


**Figure S5.** The most significantly disturbed functional network in germ-free mice model resulting from Ingenuity Pathway Analysis software.

Proteins in red were up-regulated phosphorylation in specific residues while those in green were down-regulated in germ-free (GF) compared with specific-pathogen-free (SPF) mice.


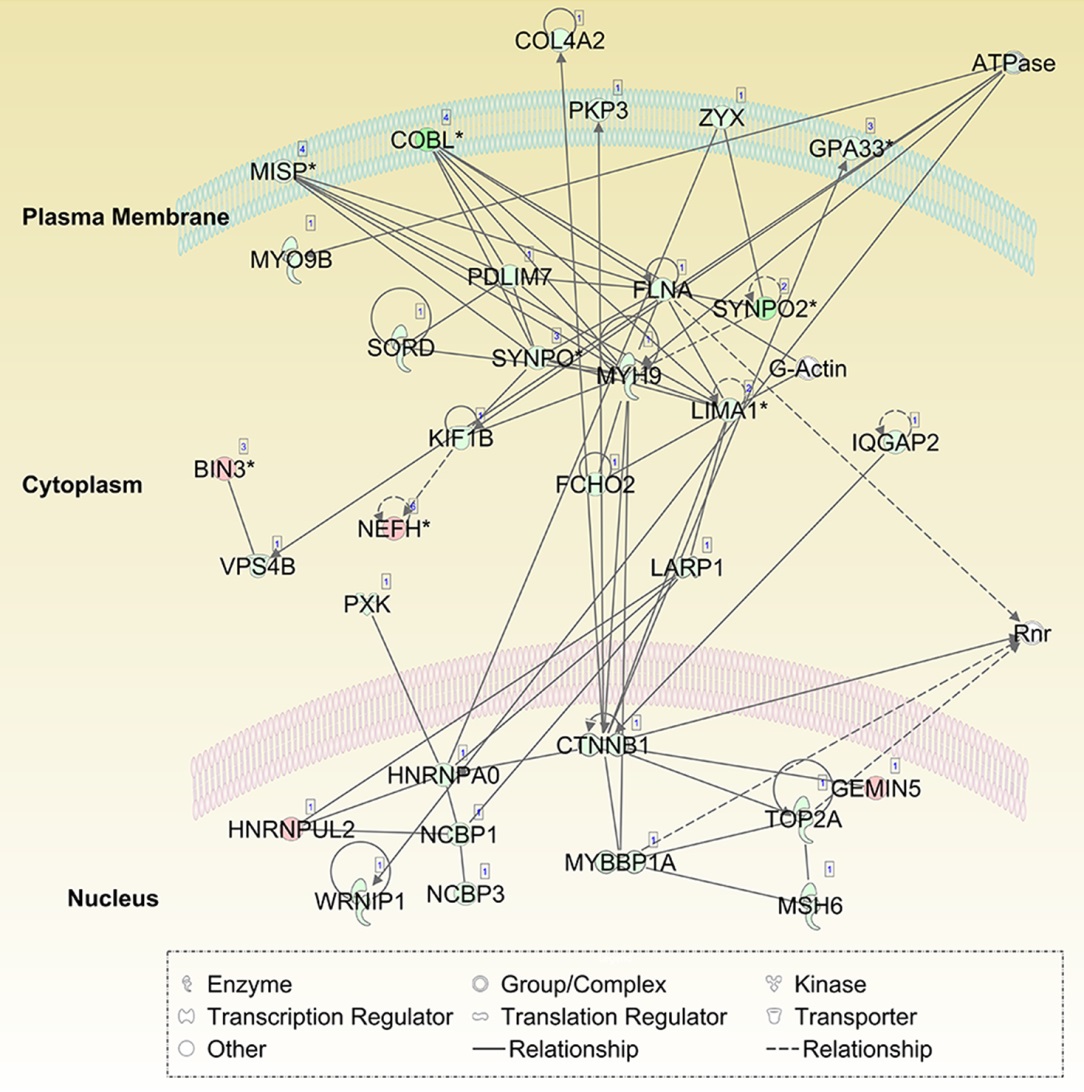


**Figure S6.** The most significantly disturbed functional network in FMT model resulting from Ingenuity Pathway Analysis software.

Proteins in red were up-regulated phosphorylation in specific residues while those in green were down-regulated in ‘depression microbiota’ recipient mice compared with ‘healthy microbiota’ recipient mice.
